# Supplementary material for: Highly sensitive and broadband meta-mechanoreceptor via mechanical frequency-division multiplexing
Source: Nat Commun. 2023 Sep 7;14:5482. doi: 10.1038/s41467-023-41222-9 (PMC10482866; doi:10.1038/s41467-023-41222-9)
Supplement: Supplementary file 1 — Supplementary Information [file 41467_2023_41222_MOESM1_ESM.pdf]

Highly sensitive and broadband meta-mechanoreceptor via mechanical frequency-division multiplexing

Authors: *Chong Li*[1], *Xinxin Liao*[1], *Zhi-Ke Peng*[1,2], *Guang Meng*[1], *Qingbo He*[1,*]

Author Affiliations:

[1]*State Key Laboratory of Mechanical System and Vibration, Shanghai Jiao Tong University, Shanghai 200240, P.R. China*

[2]*School of Mechanical Engineering, Ningxia University, Yinchuan 750021, P.R. China*

***Corresponding author**: Q. He, Email: qbhe@sjtu.edu.cn

This supporting file includes:

Supplementary Notes 1 to 19

Supplementary Figures 1 to 27

Supplementary Tables 1 to 5

Supplementary References 1 to 34

Supplemental information includes:

Supplementary Note 1. Derivation of frequency-dependent effective mass and piezoelectric coefficient

Supplementary Note 2. Details of structural parametric design in MMR

Supplementary Note 3. Details of the material property in MMR

Supplementary Note 4. Details of the mono response of MMR in d_{33} direction

Supplementary Note 5. Derivation of the mechanical frequency-division multiplexing system in MMR

Supplementary Note 6. Details of computational multi-channel demodulation for signal reconstruction

Supplementary Note 7. Details of experiments for performance verification

Supplementary Note 8. Details of the detection limit of MMR

Supplementary Note 9. Details of stability and reliability under long-time measurement and different SNRs

Supplementary Note 10. Comparison of the sensing performance of MMR with other methods

Supplementary Note 11. Details of MMR-based intelligent sensing system.

Supplementary Note 12. Details of spatio-temporal sensing

Supplementary Note 13. Recognition accuracy under different SNRs with and without MMR

Supplementary Note 14. Details of remote-vibration monitoring

Supplementary Note 15. Details of smart-driving assistance

Supplementary Note 16. Details of structural health monitoring

Supplementary Note 17. Details of running status detection of rotor test bench

Supplementary Note 18. Details of stability and reliability in different application scenarios

Supplementary Note 19. Integrated sensing of MMR

Supplementary Fig. 1 | Frequency-dependent effective piezoelectric coefficient and parameter study.

Supplementary Fig. 2 | Study on mechanical and physical properties.

Supplementary Fig. 3 | Response simulation of unit cell under excitation in different directions.

Supplementary Fig. 4 | Response details of unit cell in different directions.

Supplementary Fig. 5 | Dynamical model of the mechanical frequency-division multiplexing system.

Supplementary Fig. 6 | Bidirectional expansion of working frequency band in MMR.

Supplementary Fig. 7 | Experiments for the performance verification of MMR.

Supplementary Fig. 8 | Signal reconstruction with computational multi-channel demodulation.

Supplementary Fig. 9 | Comparison between the results measured with and without MMR.

Supplementary Fig. 10 | Detection limit study.

Supplementary Fig. 11 | Stability and reliability under long-time measurement.

Supplementary Fig. 12 | Stability and reliability at different signal-to-noise ratios (SNRs).

Supplementary Fig. 13 | Illustration of artificial deep neural network.

Supplementary Fig. 14 | Details of signal measurements in spatio-temporal sensing.

Supplementary Fig. 15 | Recognition comparison with and without MMR in spatio-temporal sensing.

Supplementary Fig. 16 | Comparison of recognition accuracy under different SNRs with and without MMR.

Supplementary Fig. 17 | Improvement of recognition accuracy by increasing training samples.

Supplementary Fig. 18 | Details of signal measurements in remote-vibration monitoring.

Supplementary Fig. 19 | Recognition comparison with and without MMR in remote-vibration monitoring.

Supplementary Fig. 20 | Details of signal measurements in smart-driving assistance.

Supplementary Fig. 21 | Recognition comparison with and without MMR in smart-driving assistance.

Supplementary Fig. 22 | Demonstration of structural health monitoring.

Supplementary Fig. 23 | Details of signal measurements in structural health monitoring.

Supplementary Fig. 24 | Recognition comparison with and without MMR in structural health monitoring.

Supplementary Fig. 25 | Running status detection of rotor test bench.

Supplementary Fig. 26 | Specific data on stability and reliability in different application scenarios.

Supplementary Fig. 27 | Integrated sensing of MMR.

Supplementary Table 1. Structural parameters of the designed unit cells in MMR.

Supplementary Table 2. Dynamic parameters of the mechanical frequency-division multiplexing system.

Supplementary Table 3. Comparisons of bandwidth between the resonant sensors and our work

Supplementary Table 4. Comparisons between the bioinspired sensors and our work

Supplementary Table 5. Frequency parameters of signal components in spatio-temporal sensing.

Supplementary text

Supplementary Note 1. Derivation of frequency-dependent effective mass and piezoelectric coefficient

Metamaterials have effective properties (e.g. dynamic effective mass) that are beyond the naturally occurring structures[1,2]. We derive the frequency-dependent effective piezoelectric coefficient $d_{33\mathrm{eff}}(\omega)$ in conjunction with the frequency-dependent effective mass $m_{\mathrm{eff}}(\omega)$ in the unit cell. Here, the $d_{33\mathrm{eff}}(\omega)$ is defined on the basis of elastodynamics theory[3,4] to describe the correlation between the stress and the electric displacement of the unit cell at different frequencies, surpassing the limitations of intrinsic piezoelectric materials[5] in which the existing piezoelectric tensors are defined under quasi-static conditions.

We present the model of a unit cell as shown in **Supplementary Fig. 1a** and **Supplementary Fig. 1b**. When MMR measures the micro-motion excitation F in the z-direction, each unit cell can be simplified as a resonator by dynamics modelling as shown in **Supplementary Fig. 1c**. Here, we define the masses of the spiral base, the piezoelectric stack and the copper pillar in the unit cell as M, m_p and m_c, respectively. For the piezoelectric stack, the piezoelectric tensor and capacitance are d_{33} and C_0, respectively. Since the connection stiffness of the piezoelectric stack and copper pillar is very large, the mass m_p and mass m_c are regarded as a center mass m ($m = m_p + m_c$) together. And the spiral beam with θ_n is equivalent to the spring stiffness k_n and damper c_n that connect the center mass m and the base mass M. When an external force F is applied to the base mass M, we defined the resultant force of spring stiffness k_n and damper c_n to the base mass as f_n^s. For the single unit cell, the displacements of m and M are expressed as $u_n^s e^{-i\omega t}$ and $U^s e^{-i\omega t}$, respectively.

Then, we derived the frequency-dependent effective mass $m_{\mathrm{eff}}(\omega)$ of the unit cell by the analytical method as follows. From Hook's law, we can get the following equation:

$$f_n^s = k_n \cdot \left(U^s - u_n^s\right) + c_n\left(-i\omega\right)\cdot\left(U^s - u_n^s\right) \tag{S1}$$

Based on Newton's law, we can get:

$$f_n^s = \left(-i\omega\right)^2 m u_n^s \tag{S2}$$

Substituting **Equation S1** into **Equation S2**, we can get:

$$u_n^s = \frac{k_n - i\cdot c_n\omega}{k_n - \omega^2 m - i\cdot c_n\omega} U^s \tag{S3}$$

Here, we defined the output charge $q_n^s(\omega)$ of the piezoelectric stack in the unit cell as

$$q_n^s(\omega) = d_{33} m_c \cdot (-i\omega)^2 u_n^s \tag{S4}$$

And we can get the output voltage $V_n^s(\omega)$ of a single unit cell as

$$V_n^s(\omega) = \frac{q_n^s(\omega)}{C_0} \tag{S5}$$

For the base mass M based on Newton's law, we can get:

$$F - f_n^s = (-i\omega)^2 MU^s \tag{S6}$$

We defined the effective mass $m_{\mathrm{eff}}(\omega)$ of one single unit cell as

$$m_{\mathrm{eff}}(\omega) = \frac{F}{-\omega^2 U^s} \tag{S7}$$

Substituting **Equations S2** and **S6** into **Equation S7**, we get:

$$F = (-i\omega)^2 \left(MU^s + mu_n^s\right) = (-i\omega)^2 m_{\mathrm{eff}}(\omega) U^s \tag{S8}$$

Substituting **Equation S3** into **Equation S8**, we get:

$$m_{\mathrm{eff}}(\omega) = M + m\frac{k_n - i\cdot c_n\omega}{k_n - \omega^2 m - i\cdot c_n\omega} \tag{S9}$$

Notably, the $m_{\mathrm{eff}}(\omega)$ was a frequency-dependent value that was close to zero at a specific frequency denoted by zero-mass frequency. Substituting **Equations S3, S7** and **S9** into **Equation S4**, we calculated $q_n^s(\omega)$ as:

$$q_n^s(\omega) = \frac{d_{33}m_c F}{m_{\mathrm{eff}}(\omega)} \cdot \frac{k_n - i\cdot c_n\omega}{k_n - \omega^2 m - i\cdot c_n\omega} = \frac{d_{33}m_c F\dfrac{k_n - i\cdot c_n\omega}{k_n - \omega^2 m - i\cdot c_n\omega}}{(M + m\dfrac{k_n - i\cdot c_n\omega}{k_n - \omega^2 m - i\cdot c_n\omega})} \tag{S10}$$

From **Equations S9** and **S10**, we found that the micro-motion response with limited excitation (finite F) was enhanced by the zero effective mass ($m_{\mathrm{eff}}(\omega) \longrightarrow 0$) with a theoretical near-infinite acceleration amplitude ($F/m_{\mathrm{eff}}(\omega) \longrightarrow \infty$). And the output charge $q_n^s(\omega)$ of the unit cell reached a near-infinite value at the zero-mass frequency. We can calculate the $d_{33\mathrm{eff}}(\omega)$ of the single unit cell, which describes the correlation between the stress and the electric displacement of the unit cell at different frequencies[6,7]

$$d_{33\mathrm{eff}}(\omega) = \frac{q_n^s(\omega)}{F} = \frac{d_{33}m_c(k_n - i\cdot c_n\omega)}{(k_n - \omega^2 m - i\cdot c_n\omega)} \cdot \frac{1}{m_{\mathrm{eff}}(\omega)} \tag{S11}$$

Here, the detailed parameter information in the above derivation process is shown in **Supplementary Table 2**. For the single unit cell ($\theta_n = 11.25$), we plotted the curves of $m_{\mathrm{eff}}(\omega)$ and $d_{33\mathrm{eff}}(\omega)$ in **Fig. 2b** of the main text, from which the $d_{33\mathrm{eff}}(\omega)$ of the unit cell appeared as a peak at the zero-mass frequency due to the presence of damping c_n. The theoretical derivations were in good agreement with the numerical simulations. Therefore, with the zero effective mass property, the $d_{33\mathrm{eff}}(\omega)$ of a single unit cell is a near-infinite value at the zero-mass frequency, which has not been demonstrated in existing piezoelectric tensors[8] and is the key characteristic for the highly sensitive micro-motion sensing.

Supplementary Note 2. Details of structural parametric design in MMR

For the structural design of MMR, we determined the structural parameters through systematic parameter design combined with the existing fabrication technology. As shown in **Supplementary**

Figs. 1a-b, the structural parameters h_1, h_2, h_3, and a of the unit cell are the height of the copper pillar, the height of the piezoelectric stack, the height of the spiral base, and the width of the spiral base, respectively. We configure the structural parameters to achieve the frequency-dependent zero effective mass. From the **Supplementary Note 1**, considering the condition of small damping, we set $m_{\text{eff}}(\omega)$ to 0 in **Supplementary Equation S9**, and the corresponding frequency ω_0 (defined as the zero-mass frequency) can be approximately described as:

$$\omega_0 = \sqrt{\frac{k_n}{m}} \cdot \sqrt{\frac{M+m}{M}} \tag{S12}$$

Since the sensor is fixed to the surface of the object to be measured, the mass M will be much greater than the m. It can be seen from the **Supplementary Equation S12** that the value of ω_0 mainly depends on k_n and m. In this design, ω_0 decreases as m increases, and ω_0 increases as k_n increases. Combined with the structure of the unit cell, h_1 and h_2 mainly determine the value of m. The value of h_3 has an influence on k_n and M. And a mainly determines the value of M. Combined with the above analytical derivation, we further use the numerical method to verify the influence of parameters.

We present the results of the parameter study in **Supplementary Figs. 1d-g**. We set the basic parameters of the unit cell as: $h_1 = 3.64$ mm, $h_2 = 5$ mm, $h_3 = 2.5$ mm, $a = 10$ mm and $\theta_n = 11.25$. First, we adjust h_1 from 1 mm to 10 mm while other parameters remain unchanged, and calculate the zero-mass frequency of the unit cell in the finite element analysis. From the numerical results as shown in **Supplementary Fig. 1d**, the ω_0 decreases with the increase of h_1, which is caused by the increase of the m due to the increase of h_1. Similarly, as shown in **Supplementary Fig. 1e**, we set h_2 from 1 mm to 10 mm, and also found that ω_0 decreases with the increase of h_2. Since the density and cross-sectional area of the copper pillar are larger than those of the piezoelectric stack, the change of h_1 has a greater influence on ω_0 than the change of h_2. Then, we set h_3 from 1 mm to 5 mm as shown in **Supplementary Fig. 1f**, and found that ω_0 increases with the increase of h_3. This is because the increase in the thickness of the helical base leads to the increase in the thickness of the helical beam, which in turn increases the stiffness k_n, resulting in an increase in the ω_0. Finally, we set a from 5 mm to 10 mm as shown in **Supplementary Fig. 1g**, and found that ω_0 changes slightly with the change of a. This is because an increase in a only causes an increase in M and no change in stiffness k_n. Combined with the above analysis of **Supplementary Equation S12**, the change of M has little effect on the change of ω_0.

Essentially, the realization of zero-mass frequency ω_0 is the key to MMR design. We have systematically studied the effects of h_1, h_2, h_3 and a on the ω_0. Our results are as follows: ω_0 decreases rapidly with the increase of h_1; ω_0 decreases slowly with the increase of h_2; ω_0 increases significantly with the increase of h_3; ω_0 approximately remains constant with the increase of a. Moreover, there is another parameter θ_n that has a greater impact on ω_0. As shown in Fig. 2c of the main text, ω_0 decreases significantly as θ_n increases. For MMR, we design the structural parameters considering the fabrication method and cost. We first fabricated the spiral base of the unit cell by 3D printing photosensitive resin. In order to ensure the processing accuracy and strength of the

spiral beam while keeping the size as small as possible, we design the parameters as h_3 = 2.5 mm and a = 10 mm. As shown in **Supplementary Fig. 1b**, the maximum range of θ_n can be 8.0 to 17.5 in a square base of 10 mm by 10 mm. Then, for copper pillars and piezoelectric stacks, we design parameters as follows in combination with current commercial machining and sintering processes: h_1 = 3.64 mm and h_2 = 5 mm. In such a structural design, the maximum sensitivity of MMR is improved by two orders of magnitude compared to conventional mechanics-guided mechanoreceptors, and its bandwidth with ultrahigh sensitivity is extendable towards both low-frequency and high-frequency ranges in 0-12 kHz through tuning the local resonance of each individual sensing cell.

In summary, we design the parameters of the unit cell as: h_1 = 3.64 mm, h_2 = 5 mm, h_3 = 2.5 mm and a = 10 mm. The range of the spiral angle is 8.0 to 17.5, which is to realize distributed zero effective masses at different frequencies. These structural parameters are determined through systematic study combined with the existing fabrication technology, which is close to the optimal design.

Supplementary Note 3. Details of the material property in MMR

We further analyzed the effect of material properties on the zero-mass frequency ω_0. From **Supplementary Note 2**, combined with the existing mature fabrication technology, the materials of the copper pillar and the piezoelectric stack are fabricated by metal copper and piezoelectric ceramic transducer (PZT) respectively. The mechanical properties of the copper pillars and the piezoelectric stack mainly affect the central mass m of the resonator and have no effect on the stiffness k_n as shown in **Supplementary Equation S12**. Compared with the central mass adjustment, the stiffness adjustment is more efficient in regulating the ω_0 in terms of cost reduction, fabrication simplification and size reduction. For MMR, our design is to change k_n by adjusting the structural parameter θ_n of the spiral base of the unit cell, thus adjusting the ω_0. Next, we further selected four main material properties for analysis: three mechanical properties are elastic modulus E, shear modulus G and density ρ of the material used in the spiral base. Another physical property is the relative dielectric constant ε of the piezoelectric stack.

We present the results of the material property study in **Supplementary Fig. 2a-d**. Here we change the shear modulus G by adjusting Poisson's ratio μ, where $G = E/2(1+\mu)$ is satisfied. We set the basic parameters of the unit cell as: $E = 2.5\times10^9$ Pa, μ = 0.41, ρ = 1250 kg/m^3 and ε = 1433.6. First, we adjust E from 1×10^9 Pa to 10×10^9 Pa while other parameters remain unchanged, and calculate the zero-mass frequency ω_0 of the unit cell in the finite element analysis. From the numerical results as shown in **Supplementary Fig. 2a**, the ω_0 increases with the increase of E, which is caused by the increase of the k_n due to the increase of E. Then, we set μ from 0.3 to 0.45 as shown in **Supplementary Fig. 2b**, and found that ω_0 remains approximately unchanged with the increase of μ. This is because an increase in μ has no change in stiffness k_n, which also means that only changes in the shear modulus have no effect on the stiffness k_n. Similarly, as shown in **Supplementary Fig. 2c**, we set ρ from 800 kg/m^3 to 1700 kg/m^3, and also found that ω_0 remains

approximately unchanged with the increase of ρ. This is because an increase in ρ only causes an increase in M and no change in k_n. Combined with the analysis of **Supplementary Equation S12**, the change of M has little effect on the change of ω_0. Finally, we set ε from 1000 to 2000 as shown in **Supplementary Fig. 2d**, and found that ω_0 still remains approximately unchanged with the change of ε. This is because the variation of the dielectric constant of the piezoelectric stack has no effect on the mechanical properties of the unit cell with mechanics-guided design.

From the results shown in **Supplementary Fig. 2**, we have systematically studied the effects of E, μ, ρ and ε on the ω_0. Our results are as follows: ω_0 increases significantly with the increase of E; ω_0 remains approximately unchanged with the change of μ, ρ and ε. For material's mechanical properties, this means that the change in the elastic modulus causes a change in stiffness and thus changes the zero-mass frequency. The changes in the shear modulus and density of the helical base have no effect on the zero effective mass. Considering the design of zero-mass frequency in the kilohertz frequency band, we choose non-metal as the material of the spiral base, so that the zero-mass design can be completed with a smaller θ_n (from 8.0 to 17.5) to reduce the overall structure size. If the metal is used as the base material, such as iron, the 10 times larger θ_n is required to design a bandwidth of 0-12 kHz. The large θ_n will enlarge the overall structure size of the MMR, and the difficulty of manufacturing the metal helix makes the entire fabrication process very complicated. After comprehensive consideration, we selected the photosensitive resin (DSM IMAGE8000) from Royal DSM to make all the spiral substrates by 3D printing, which can control the fabrication error within 0.1 mm. Since DSM IMAGE8000 has mature fabrication technology, small fabrication error and low fabrication cost, the material selection for the spiral base is close to optimal combined with the existing fabrication technology. For material's physical properties, the dielectric constant of the piezoelectric stack has no effect on the mechanical properties of the MMR. The piezoelectric stack only acts as a transducer in the MMR. The choice of dielectric constant is not the focus of our work because our work focuses on a mechanics-guided design. Therefore, we selected the most commonly used PZT for the material selection of the piezoelectric stack which has high signal output, stable working performance and mature fabrication technology.

In summary, we have further systematically analyzed the effect of the materials' mechanical properties and physical properties on the zero effective mass. A change in its elastic modulus causes a change in stiffness and thus changes the zero-mass frequency. The changes in the shear modulus and density of the helical base have no effect on the zero effective mass. The dielectric constant of the piezoelectric stack has no effect on the mechanical properties of the MMR. We selected the photosensitive resin (DSM IMAGE8000) to make the spiral bases by 3D printing. We selected the commonly used PZT to fabricate the piezoelectric stack. We chose standardized metal copper to make copper pillars. We set the material parameters of photosensitive resin, piezoelectric stack and copper pillar as follows: E_{res} = 2.5 Gpa, E_{pie} = 117.4 Gpa and E_{cop} = 110.0 Gpa; shear modulus G_{res} = 1.025 Gpa, G_{pie} = 23.5 Gpa and G_{cop} = 38.5 Gpa; mass density ρ_{res} = 1250 Kg m^{-3}, ρ_{pie} = 7500 Kg m^{-3} and ρ_{cop} = 8960 Kg m^{-3}. The dielectric constant of the piezoelectric stack was set to 1433.6. These material properties are determined through systematic study combined with the existing fabrication technology, which is close to the optimal design.

Supplementary Note 4. Details of the mono response of MMR in d_{33} direction

Since the practical micro-motions are multi-directional, we further give the explanation to approve MMR is mono responded in d_{33} direction as shown in **Supplementary Fig. 3**. Here, d_{33} describes the correlation between the stress and the electric displacement in the intrinsic piezoelectric material along the z-direction in Cartesian coordinates. As shown in **Supplementary Fig. 3a**, the piezoelectric stack is between the spiral base and the copper pillar, and converts the micro-motion excitation from the spiral base into a voltage signal. The piezoelectric stack adopts the z-direction stacking manufacturing process, and we have tested that only the z-direction charge output is generated. Since the piezoelectric stack is installed on the helical base, applying weak vibration excitations in different directions to the helical base can only cause the piezoelectric stack to produce voltage outputs in the z-direction. Here, the micro-motions in different directions can cause the stress or shear force in different directions in the spiral base. As shown in **Supplementary Fig. 3b**, we list all 6 possible micro-motion directions applied to the spiral base, including stress direction along x axis (denoted as 1), stress direction along y axis (denoted as 2), stress direction along z axis (denoted as 3), shear force direction rotating around x axis (denoted as 4), shear force direction rotating around y axis (denoted as 5) and shear force direction rotating around z axis (denoted as 6). Since the nth unit cell only has voltage output in the z-direction (denoted as 3), only six directions between d_{11}^{n} and d_{66}^{n} are valid for MMR, and the six directions are d_{31}^{n}, d_{32}^{n}, d_{33}^{n}, d_{34}^{n}, d_{35}^{n} and d_{36}^{n}, respectively. We present the numerical simulations of six different directions with finite element analysis method as shown in **Supplementary Figs. 3c-h**, respectively. We apply swept-frequency micro-motion excitations of equal amplitude and different directions to the spiral base, and then calculate the voltage output of the unit cell in the z-direction.

As shown in **Supplementary Fig. 4**, we present voltage output details for different directions and normalize the output amplitude of the voltage. Taking the d_{33}^{n} direction as the normalized benchmark, the maximum output amplitude value of d_{33}^{n} is 1, while the maximum output amplitude values in the d_{31}^{n}, d_{32}^{n}, d_{34}^{n}, d_{35}^{n} and d_{36}^{n} directions are 2.2e-3, 2.05e-3, 1.4e-3, 1.8e-3 and 8e-3, respectively. From the results, the voltage output in the other five directions is far smaller than the voltage output in the direction of d_{33}, which can be approximated that MMR is mono responded in d_{33} direction.

Supplementary Note 5. Derivation of the mechanical frequency-division multiplexing system in MMR

For the developed mechanical frequency-division multiplexing system, we further derived the frequency-dependent effective mass $M_{\mathrm{eff}}(\omega)$ of the whole MMR and the effective piezoelectric coefficient $d_{33\mathrm{eff}}^{n}(\omega)$ of the nth unit cell. When MMR measures the micro-motion excitation F in the z-direction as shown in **Supplementary Fig. 5a**, the MMR can be simplified as a global dynamical model as shown in **Supplementary Fig. 5b**. Considering that MMR is assembled by N piezoelectric resonators, we rigidly connected the bases of N resonators together to form a whole mass NM. Similar to the above derivation in **Supplementary Note 1**, the displacements of m and

NM were expressed as $u_n e^{-i\omega t}$ and $Ue^{-i\omega t}$, respectively. With the external micro-motion excitation F applied to the base mass NM, we defined the resultant force of spring k_n and damper c_n to the base mass as f_n. Other parameter definitions were consistent with those in **Supplementary Note 1**. For the nth unit cell, we can get the following equation based on Hook's law:

$$f_n = k_n \cdot \left(U - u_n\right) + c_n\left(-i\omega\right)\cdot\left(U - u_n\right) \tag{S13}$$

Based on Newton's law, we can get:

$$f_n = \left(-i\omega\right)^2 mu_n \tag{S14}$$

Substituting **Equation S13** into **Equation S14**, we can get:

$$u_n = \frac{k_n - i\cdot c_n\omega}{k_n - \omega^2 m - i\cdot c_n\omega}U \tag{S15}$$

Here, we defined the output charge $q_n(\omega)$ of the piezoelectric stack in the nth unit cell as

$$q_n(\omega) = d_{33}m_c \cdot (-i\omega)^2 u_n \tag{S16}$$

And we can get the output voltage $V_n(\omega)$ of the nth unit cell as

$$V_n(\omega) = \frac{q_n(\omega)}{C_0} \tag{S17}$$

For the base mass NM based on Newton's law, we can get:

$$F - \sum_{n=1}^{N} f_n = \left(-i\omega\right)^2 NMU \tag{S18}$$

We defined the effective mass $M_{\text{eff}}(\omega)$ of the whole dynamical model as

$$M_{\text{eff}}(\omega) = \frac{F}{-\omega^2 U} \tag{S19}$$

Substituting **Equations S14** and **S18** into **Equation S19**, we get:

$$F = \left(-i\omega\right)^2\left(NMU + \sum_{n=1}^{N} mu_n\right) = \left(-i\omega\right)^2 M_{\text{eff}}(\omega)U \tag{S20}$$

Substituting **Equation S15** into **Equation S20**, we get:

$$M_{\text{eff}}(\omega) = NM + \sum_{n=1}^{N} m\frac{k_n - i\cdot c_n\omega}{k_n - \omega^2 m - i\cdot c_n\omega} \tag{S21}$$

Here, the M_{eff} has distributed zero effective masses in different frequencies. Combining **Equations S15, S16, S19, S20** and **S21**, we can obtain the effective piezoelectric charge coefficient $d_{33\text{eff}}^{n}(\omega)$ of the nth unit cell as

$$d_{33\text{eff}}^{n}(\omega) = \frac{q_n(\omega)}{F} = \frac{d_{33}m_c(k_n - i\cdot c_n\omega)}{(k_n - \omega^2 m - i\cdot c_n\omega)}\cdot\frac{1}{M_{\text{eff}}(\omega)} \tag{S22}$$

Here, the detailed parameter information in the above derivation is shown in **Supplementary Table 2**. We plotted the curves of $M_{\text{eff}}(\omega)$ and $d^{n}_{33\text{eff}}(\omega)$ (n = 1,2, … ,9) in **Supplementary Fig. 5c**, from which the $d^{n}_{33\text{eff}}(\omega)$ appeared as multiple peaks at the distributed zero-mass frequencies due to the small damping c_n. The analytical results with theoretical derivation were in good agreement with the experimental and numerical results shown in **Fig. 2f** of the main text, in which we constructed MMR with 3×3 unit cells in the selected frequency band of 0-3.5 kHz. To further demonstrate the bidirectional expansion of the bandwidth, we extended the MMR to 4×4 unit cells based on the original 3×3 unit cells denoted by the white box as shown in **Supplementary Fig. 6a**. We demonstrated that the frequency band Δb_n allowed for a tailored design of center zero-mass frequency in 0-12 kHz by individually tuning the parameter θ_n of the unit cell as shown in **Supplementary Fig. 6b**. By designing distributed zero effective masses in different frequencies, MMR exhibited frequency-dependent effective piezoelectric coefficients with enhanced micro-motion sensing in non-overlapping frequency bands, which collectively constituted a mechanical frequency-division multiplexing system, providing as a working mechanism for highly sensitive and broadband micro-motion sensing.

Supplementary Note 6. Details of computational multi-channel demodulation for signal reconstruction

We constructed a micro-motion sensing system based on the as-fabricated MMR and presented the schematic diagram of the experimental layout as shown in **Supplementary Fig. 7**. We presented the detailed workflow of computational multi-channel demodulation as shown in **Supplementary Fig. 8**. During the working process of MMR, each unit cell was a narrowband measurement channel with piezoelectric voltage output, which modulated the micro-motion response nonlinearly in a specific frequency range. Here, we described how we reconstructed the unknown micro-motion excitation $s(t)$ from the measured data including the frequency responses $H_n(\omega)$ and piezoelectric voltage signals $v_n(t)$. Combining **Equations S16** and **S22** of **Supplementary Note 5**, we measured the voltage signal in each channel, and the $H_n(\omega)$ satisfied

$$H_n(\omega) = \frac{d^{n}_{33\text{eff}}(\omega)}{C_0} \qquad (n = 1, 2, 3, ..., N) \tag{S23}$$

where C_0 is the capacitance of the employed piezoelectric stack in MMR. For the nth measurement channel, the measured data satisfied:

$$S(\omega)H_n(\omega) = V_n(\omega) \qquad (n = 1, 2, 3, ..., N) \tag{S24}$$

where $S(\omega)$ and $V_n(\omega)$ are the frequency-domain representations of the signals $s(t)$ and $v_n(t)$. Here, we defined the lower and upper limits of the working frequency band of MMR as $\omega_{\min}$ and $\omega_{\max}$, respectively. According to the specific frequency response $H_n(\omega)$ of each channel, we divided the frequency band into N parts by calculating the following equation:

$$H_{n-1}(\omega_n) = H_n(\omega_n) \qquad (2 \le n \le N) \tag{S25}$$

We defined the $\omega_{\min}$ and $\omega_{\max}$ as the ω_1 and ω_{N+1}, respectively. Thus, with **Equation S25**, we can

get a group of ω as:

$$\omega = \left[\omega_1, \omega_2, ..., \omega_{N+1}\right] \tag{S26}$$

Then we determined the working frequency band of the nth channel as:

$$H_n^{'}(\omega) = H_n(\omega) \qquad (\omega_n \le \omega \le \omega_{n+1}) \tag{S27}$$

Next, we used a least-mean-square adaptive filter algorithm to process the measured voltage $v_n(t)$ of the nth channel and then performed Fourier transform algorithm to obtain the $V_n(\omega)$. Similarly, we calculated the corresponding voltage signals of the nth measurement channel at the working frequency band as:

$$V_n^{'}(\omega) = V_n(\omega) \qquad (\omega_n \le \omega \le \omega_{n+1}) \tag{S28}$$

Combining **Equations S27** and **S28**, we defined the frequency-domain representation $S_n^{'}(\omega)$ of the signal transmitted by the nth channel as:

$$S_n^{'}(\omega) = \frac{V_n^{'}(\omega)}{H_n^{'}(\omega)} \qquad (\omega_n \le \omega \le \omega_{n+1}) \tag{S29}$$

We calculated the inverse Fourier transform (IFT) of $S_n^{'}(\omega)$ as:

$$s_n^{'}(t) = IFT\left[S_n^{'}(\omega)\right] \qquad (\omega_n \le \omega \le \omega_{n+1}) \tag{S30}$$

Then, we can get the reconstructed vibration signal as:

$$s(t) = \sum_{n=1}^{N} s_n^{'}(t) \tag{S31}$$

Last, we can obtain the time-frequency representation of the reconstructed signal $s(t)$ by performing the short-time Fourier transform (STFT) algorithm:

$$S(t,\omega) = STFT\left[s(t)\right] \qquad (\omega_1 \le \omega \le \omega_{N+1}) \tag{S32}$$

From the workflow of the signal reconstruction process, a broadband range of micro-motion induced voltage output was measured simultaneously through N channels, which was further reconstructed with a computational multi-channel demodulation approach. This method is to deal with the nonlinear modulation effect of the micro-motion response in the mechanical frequency-division multiplexing system, which directly determines the accuracy of signal reconstruction.

Supplementary Note 7. Details of experiments for performance verification

We presented the measurement details as shown in **Supplementary Fig. 9**. For the original multi-frequency signal, we constructed nine harmonic components of equal amplitude. The frequency values of nine harmonic components were around 437 Hz, 684 Hz, 964 Hz, 1250 Hz, 1540 Hz, 1900 Hz, 2130 Hz, 2480 Hz and 2880 Hz, respectively. We added background noise to the original multi-frequency signal to construct an experimental multi-frequency signal with an SNR of -20 dB.

With the above workflow of signal reconstruction as shown in **Supplementary Fig. 8**, we obtained the time-frequency representation of the experimental multi-frequency signal with MMR. From the reconstructed signal spectrum with MMR as shown in **Supplementary Fig. 9a**, the measured result presented a clear time-frequency representation of multi-frequency components. In contrast, as shown in **Supplementary Fig. 9b**, the result with conventional piezoelectric mechanoreceptor presented unclear signal components and strong background noise, because the multi-frequency components with an SNR of -20 dB cannot reach the detection limit of conventional piezoelectric mechanoreceptor.

We further presented the time-domain details of the measured signal. As shown in **Supplementary Fig. 9c**, each harmonic component appeared sequentially at 0.1 s intervals in the multi-frequency signal denoted by S_1. The measured result that was denoted by S_2 without MMR was shown in **Supplementary Fig. 9d**, from which we found that the measured signal contained strong background noise. Through computational sensing, we presented the measured result denoted by S_o with MMR in **Supplementary Fig. 9e**. We obtained the measurement error (S_o-S_1) from 0.450 s to 0.475 s as shown in **Supplementary Fig. 9f**. The average measurement error was less than 3%, which was calculated by the amplitude ratio of the error to the original signal. Furthermore, we improve the accuracy of the testing data by averaging multiple repeated measurements. As shown in **Supplementary Fig. 9g**, we presented the measured result denoted by S_m with MMR via five repeated measurements. We obtained the measurement error (S_m-S_1) from 0.450 s to 0.475 s as shown in **Supplementary Fig. 9h**. The average measurement error via five repeated measurements was less than 1%. As a result, we demonstrate that the reconstruction accuracy of measured data can be further improved by repeated measurements. Compared with the one-time measurement, the measurement error of five repeated measurements with computational sensing is reduced from 3% to 1% in this demonstrated case. From the measured results and error analysis, we found that the measured results with MMR were relatively close to the original signal even in an SNR of -20 dB. Thus, we achieved highly sensitive and broadband micro-motion sensing in MMR that is inaccessible in the conventional piezoelectric mechanoreceptor.

Supplementary Note 8. Details of the detection limit of MMR

In our experiments, we use the physical quantity of acceleration to characterize the magnitude of the micro-motion stimulation, and have calculated that MMR has a maximum sensitivity of 36.540 mv $m^{-1}s^2$ in the main text. Because the charge output of MMR is the largest at 1540 Hz as shown in **Fig. 2f** of the main text, we applied a single-frequency excitation of 1540Hz with different strengths to MMR as shown in **Supplementary Fig. 10a**. Then, we estimate the detection limit by extrapolating the data from **Supplementary Fig. 10b**. As shown in **Supplementary Fig. 10a**, we obtained that when the micro-motion stimulation is 3.6×10^{-2} g and 3.6×10^{-3} g, the corresponding voltage outputs are 12.89 mv and 1.29 mv, respectively. At the same time, a signal with the smallest amplitude of 1×10^{-4} mv may be detected, corresponding to a detection level of 0.28×10^{-6} g as shown in **Supplementary Fig. 10b**. For comparison, we further decreased the intensity of micro-motion stimulation, and then observed the voltage output of MMR. As shown in **Supplementary Fig. 10c**,

we obtained that when the micro-motion stimulation is 6.0×10^{-4} g and 6.0×10^{-5} g, the corresponding voltage outputs are 0.21 mv and 0.02 mv, respectively. At the same time, a signal with the smallest amplitude of 1×10^{-4} mv can also be detected, corresponding to a detection level of 0.28×10^{-6} g as shown in **Supplementary Fig. 10d**. By comparing **Supplementary Figs. 10b** and **10d**, we found that the signal with minimum amplitude of 1×10^{-4} mv can both be observed under different intensities of micro-motion stimulation, which verifies that the lowest detection limit can reach 0.28×10^{-6} g. Thus, the lowest detection limit is estimated to reach 10^{-6} g in the order of magnitude.

Supplementary Note 9. Details of stability and reliability under long-time measurement and different SNRs

Since the MMR structure has strong stability below 65°C, the temperature change during the measurement at room temperature has little effect on the sensing performance. We mainly verified the stability and reliability of MMR through experiments under long-time measurement and under measurement of different SNRs. **First**, considering that the sensor needs to be calibrated before working, we study the amplitude stability of the sensor during a continuous long-term measurement. Since MMR is composed of multiple unit cells, we take the first unit cell (zero-mass frequency of 436 Hz) as an example to analyze its stability and reliability. As shown in **Supplementary Fig. 11a**, we firstly apply a swept-frequency micro-motion excitation to the MMR from 0 Hz to 3500 Hz at the room temperature of 25°C, and the excitation time of a single cycle is 10 seconds. From the output results (with normalized amplitude) of the unit cell, it can be seen that the unit cell has a strong output at about 1.25 s because the frequency of the excitation signal at this time is near the zero-mass frequency of the MMR. And the average value of the whole output signal does not deviate from the zero starting point, which means that the zero drift of the average amplitude is small. As shown in **Supplementary Fig. 11b**, to further quantify these zero drifts of average amplitude, we applied a cyclic swept-frequency excitation to the MMR over 10 cycles with a duration of 100 s. We then calculated the zero drift of the MMR under 10 cycles of excitation as shown in **Supplementary Fig. 11c**. From the calculation results, the MMR has a slight zero drift with a periodic amplitude less than 0.01 under the long-term cyclic excitation. This may be due to the low-frequency disturbance oscillation caused by the sweep frequency excitation, which can be eliminated and calibrated by a simple filtering algorithm in the computational multi-channel demodulation process.

Second, we analyzed the amplitude stability of the unit cell when it is near resonance. We applied a 436 Hz harmonic excitation to the MMR and measured the amplitude output of the first unit cell. The details of the amplitude output are shown in **Supplementary Fig. 11d**. We measured the harmonic excitation continuously for 1000 s from the start operation with MMR at 25°C. The results of continuous measurement are shown in **Supplementary Fig. 11e**. Due to the unavoidable damping, MMR showed a stable resonant output, which resembled an enhanced forced oscillation with a stable output. When conducting experiments, we waited for the MMR to operate for about 20 s and started collecting data after the output is stable. We further calculated the maximum absolute value of the zero drift amplitude within 1000 seconds as shown in **Supplementary Fig.**

11f. From the calculated results, the zero drift is less than 0.01. This zero-drift can be corrected by the post-processing algorithm.

Third, we analyzed the stability and reliability of MMR at different signal-to-noise ratios (SNRs). As shown in **Supplementary Fig. 12**, we conducted comparative experiments with a chirp signal as the broadband signal with SNR of 10 dB, 5 dB, 0 dB, -10 dB, -20 dB and -30 dB, respectively. We used the MMR and the conventional piezoelectric mechanoreceptor to measure micro-motion signals under different signal-to-noise ratios and make comparative analysis. The conventional piezoelectric mechanoreceptor here is a piezoelectric accelerometer with non-resonant sensor design. We use the conventional piezoelectric mechanoreceptor as a measurement reference to analyze the measurement performance of MMR at different signal-to-noise ratios. From the measured results as shown in **Supplementary Figs. 12a-b**, when the SNR is 10 dB and 5 dB, both the MMR and the conventional piezoelectric mechanoreceptor can measure clear and similar time-varying frequency components. When the SNR is 0 dB, the measured result with MMR presented clearer time-varying frequency components than that with the conventional piezoelectric mechanoreceptor as shown in **Supplementary Fig. 12c**. When the SNR is -10 dB, MMR can still measure clear time-varying frequency components, but the measurement results from conventional piezoelectric mechanoreceptors show strong background noise as shown in **Supplementary Fig. 12d**. Moreover, when the SNR reaches -20 dB and -30 dB as shown in **Supplementary Figs. 12e-f**, the MMR can still measure the majority of the signal components, while the conventional piezoelectric mechanoreceptor cannot measure clear signal components since the chirp signal with SNR of -20 dB and -30 dB cannot reach the detection limit. As a result, under different SNRs, MMR is superior to conventional piezoelectric mechanoreceptors in micro-motion sensing, and has strong stability and reliability in sensing performance.

In summary, we further demonstrated that MMR shows strong stability and reliability under long-time measurement of 1000 s and under different SNRs from -30 dB to 10 dB.

Supplementary Note 10. Comparison of the sensing performance of MMR with other methods

We further compared our work with other methods in terms of sensitivity and bandwidth. **First,** the high sensitivity of the MMR is due to the frequency-dependent effective piezoelectric coefficient $d_{33\mathrm{eff}}(\omega)$ generated by the zero effective masses. Here, d_{33} describes the correlation between the stress and the electric displacement in the intrinsic piezoelectric material along the z-direction in Cartesian coordinates. The relationship between the sensitivity $S(\omega)$ and $d_{33\mathrm{eff}}(\omega)$ is:

$$S(\omega)=Cd_{33\mathrm{eff}}(\omega) \qquad \text{(S33)}$$

where C is a positive constant coefficient. Generally, the sensitivity increases with the increase of $d_{33\mathrm{eff}}(\omega)$, and we use $d_{33\mathrm{eff}}(\omega)$ as an indicator to compare the sensitivity of different sensor designs. Here, we define $d_{33\mathrm{eff}}(\omega)$ based upon elastodynamics theory to describe the electromechanical conversion of the unit cell in different frequencies as follows

$$d_{33\mathrm{eff}}(\omega)=A(\omega)d_{33}\frac{1}{m_{\mathrm{eff}}(\omega)} \quad \text{(S34)}$$

where $A(\omega)$ represents a frequency-dependent variable related to the dynamic parameter of the unit cell, and ω denotes the varying frequency. As shown in **Fig. 2b** of the main text, we found that the $d_{33\mathrm{eff}}(\omega)$ was theoretically a near-infinite value at the zero-mass frequency, which has not been demonstrated in existing piezoelectric tensors. As shown in **Fig. 2d** of the main text, some methods in Refs. [49-57] with non-resonant structural design are limited in sensitivity improvement. In conventional piezoelectric materials, the electromechanical conversion is described by existing piezoelectric tensors defined under quasi-static conditions. Due to the limitation of quasi-static conditions, most of the piezoelectric coefficients are about 700 pC N^{-1}, and only a few can reach 2100 pC N^{-1} as shown in Ref. [49]. While for MMR, the maximum $d_{33\mathrm{eff}}(\omega)$ reached an appreciable value of 24930 pC N^{-1}, which was much higher than that of the state-of-art piezoelectric materials. The MMR combines unit cells with mechanics-guided piezoelectric resonator design to bypass the limitations of intrinsic piezoelectric materials and achieve high sensitivity. Essentially, configuring zero effective mass design in the unit cell results in a near-infinite effective piezoelectric coefficient, allowing access to micro-motion sensing with a high sensitivity around the zero-mass frequency.

Second, other methods improve sensitivity through resonant structural design, but limit the increase in bandwidth. **Supplementary** Ref. [9] developed a mode-localized resonant sensor with a bandwidth of 3.5 Hz[9]. **Supplementary** Ref. [10] and **Supplementary** Ref. [11] both developed a high resolution resonant sensor with a bandwidth of 5 Hz[10,11]. **Supplementary** Ref. [12] developed a seismic-grade resonant sensor with a bandwidth of 100 Hz[12]. **Supplementary** Ref. [13] developed a differential mode-localized sensor with a bandwidth of 320 Hz[13]. **Supplementary** Refs. [14-15] developed a differential resonant sensor and a micromachined resonant sensor with a bandwidth of 500 Hz, respectively[14,15]. **Supplementary** Ref. [16] developed a nanoresonator-based sensor with a bandwidth of 1000 Hz[16]. **Supplementary** Ref. [17] developed a programmable resonant sensor with a bandwidth of 1246 Hz[17]. **Supplementary** Ref. [18] developed a resonant accelerometer based on nanomechanical piezoresistive transduction with a bandwidth of 1500 Hz[18]. The comparisons of bandwidth between other works with resonant structural design and our work are listed in **Supplementary** Table 3. It can be seen from the table that the bandwidth can reach 320 Hz through the mode-localized resonant structure design. Further, the bandwidth can reach 1.5 kHz through the structural design of the nano-resonator. While for our work, through integrating sensing units with prescribed piezoelectric coefficients, the bandwidth of MMR with high sensitivity can be extended towards both low frequencies and high frequencies in 0-12 kHz. Since MMR is a metamaterial structure composed of unit cells featuring a broad range of local resonances, the working bandwidth can be flexibly customized.

Third, there are also some bionic methods to realize acoustic sensing, vibration sensing and tactile sensing. **Supplementary** Ref. [19] developed a human cochlea-inspired acoustic sensor with the piezoelectric coefficient of 46 pC N^{-1} in a bandwidth of 1 kHz[19]. **Supplementary** Ref. [20] developed a spider crack- inspired vibration sensor with the 100-fold sensitivity improvement in 0-1000 Hz[20]. **Supplementary** Ref. [21] developed a human skin-inspired tactile sensor which can

achieve a detection limit of 0.1 kPa with a bandwidth of 1 kHz[21]. **Supplementary** Ref. [22] developed a chameleon skin-inspired vibration sensor, and its sensitivity was measured to be the output power under 0.245% strain with a bandwidth of 5 Hz[22]. Other bionic methods focused on strain sensing and pressure sensing without consideration of the bandwidth. For example, **Supplementary** Ref. [23] developed a scorpion crack-inspired strain sensor, and its sensitivity was measured to be a Gauge factor of 1344.1 at 200% strain, which focused on the static strain sensing with no consideration of the bandwidth for dynamic micro-motion sensing[23]. Similarly, **Supplementary** Ref. [24] developed a human skin-inspired strain sensing with the 24-fold sensitivity improvement, which also ignored the bandwidth measurement[24]. Moreover, **Supplementary** Refs. [25-28] all developed the bio-inspired pressure sensors with the sensitivity of 8% Pa^{-1}, 50.17 kPa^{-1}, 8.5 kPa^{-1} and 70.86% kPa^{-1} respectively[25-28], none of which considered the measurements of bandwidth. The comparisons of bandwidth between other bionic works and our work are listed in **Supplementary Table 4**. As can be seen from the table, the biomimetic approach has inspired different sensor designs, including: acoustic sensors, vibration sensors, tactile sensors, strain sensors, and pressure sensors. Different sensors have different definitions of sensitivity, and some consider bandwidth while others do not. On the whole, these bionic methods can achieve a piezoelectric coefficient of 46 pC N^{-1}, a 24-fold increase or a 100-fold increase in sensitivity, and some of their bandwidths can reach 1 kHz. For our work, MMR can achieve a piezoelectric coefficient of 24930 pC N^{-1} and two orders of magnitude improvement in sensitivity. The MMR's bandwidth can reach 12 kHz, which can be extended towards both low frequencies and high frequencies.

In summary, we compare our work with non-resonant methods, resonant methods, and bionic methods in terms of sensitivity and bandwidth. We have reported a bioinspired MMR for highly sensitive and broadband micro-motion sensing via mechanical frequency-division multiplexing. MMR addresses the trade-off between sensitivity and bandwidth in conventional mechanics-guided micro-motion sensors. This work can provide a new perspective on simpler mechanics-guided designs of high-performance sensors for various other physical information.

Supplementary Note 11. Details of MMR-based intelligent sensing system

For the MMR-based intelligent sensing system in our experiments, the working strategy was composed of two components: in-situ signal enhancement with MMR, and signal recognition based on a deep neural network. To explain this intelligent sensing system, we presented one example as shown in **Supplementary Fig. 13a**. Here, we showed a time-frequency representation measured by MMR in a strong noise background, which was corresponding to the designed spatio-temporal pattern "S". And the time-frequency representation with MMR exhibited clear signal features, which were input into the deep neural network to complete the training and recognition algorithm, leading to high recognition accuracy. In contrast, the measurement results without MMR showed an unclear time-frequency representation due to the strong background noise as shown in **Supplementary Fig. 13b**, thus only a low recognition accuracy can be achieved.

For the employed deep neural network in this MMR-based intelligent sensing system, we presented the schematic diagram of this network as shown in **Supplementary Fig. 13c**. Here, this network was an AlexNet-based deep learning network[29], which consisted of one input layer, five convolutional layers, three fully connected layers and one output layer. First, we input the time-frequency representations of the measured signal as images to the deep neural network in the input layer. Then, the input time-frequency representations were extracted and learned by the convolutional layers, and further classified by the fully connected layers. Finally, the output layer can output the corresponding recognition results, in which we classified the results by labelling each result as #*n*. In the intelligent sensing system, MMR played a role in the in-situ enhancement of signal features, and the deep neural network was used to complete the signal training and recognition processes. In this way, the intelligent sensing system can obtain a high recognition accuracy, which has potential for perception, monitoring and identification of environmental weak vibrations.

Supplementary Note 12. Details of spatio-temporal sensing

For the demonstrated spatio-temporal sensing as shown in **Supplementary Fig. 14a**, we constructed harmonic signals of different frequencies that can sequentially generate the resonant responses of unit cells at different locations. By tracking the locations, a corresponding spatio-temporal pattern can be generated. Here, we constructed four kinds of signals consisting of different harmonic components, which corresponded to four designed patterns "SJTU". The details of signal components were listed in **Supplementary Table 5**. Then we added strong background noise to the constructed signals to achieve an SNR of -20 dB. As shown in **Supplementary Fig. 14b-e**, we presented the measured results corresponding to "S", "J", "T" and "U" with and without MMR. From the time-frequency representation results, we found that the measured results with MMR presented clearer time-frequency components than that without MMR. Thus, for the spatio-temporal sensing, the MMR played a role in the in-situ enhancement of signal features.

Then, we input the time-frequency representations with and without MMR into the deep neural network for signal recognition. For the experimental signals with an SNR of -20 dB, we constructed 400 samples, 50% of which were used for training and the other 50% for recognition. We presented the recognition details as shown in **Supplementary Fig. 15**. From the recognition results with MMR, only one sample was identified incorrectly, and the overall recognition accuracy was 99.5% as shown in **Supplementary Fig. 15a**. In contrast, from the recognition results without MMR as shown in **Supplementary Fig. 15b**, most samples were identified incorrectly and the average recognition accuracy was 34.0%, which was lower than the recognition performance with MMR.

To analyze the accuracy of identification, we presented the experimental results with an SNR of 0 dB. From the results shown in **Fig. 4b** of the main text, the recognition accuracies with and without MMR both increased when the SNR is 0 dB, but the recognition accuracy without MMR was at most 70% and was always lower than that with MMR. This result proved that although the

conventional sensor can still work at the SNR of 0 dB, its sensing performance was lower than MMR. When the SNR is -20 dB, the recognition accuracy without MMR was always below 40% even if the number of samples increased. In contrast, most of the recognition accuracies with MMR were above 90%. This result demonstrates that the conventional sensor cannot measure clear signal components because the signal has been too weak to be detected at the SNR of -20 dB, while MMR still exhibits high-performance sensing ability. Furthermore, the neural network structures with and without MMR were the same, and the single sample has approximately equal energy consumption and speed. In the training process, the whole processing speed was inversely proportional to the signal samples, and the whole energy consumption was proportional to the signal samples. Moreover, the recognition rate of 40 samples in MMR was 90%, which was still higher than the 38.8% of 400 samples without MMR. Therefore, our designed MMR held great promise to construct intelligent sensing systems with high recognition accuracy[30,31].

Supplementary Note 13. Recognition accuracy under different SNRs with and without MMR

Considering that the mechanical micro-motions can be different with different styles of vibration and energy levels, we evaluated the lowest signal-to-noise ratio (SNR) at which MMR was able to detect micro-motion signals. As shown in **Supplementary Note 11**, we constructed an intelligent sensing system by combining MMR with a deep learning technique. The micro-motion signal was measured by MMR and then was transported to a deep neural network to complete the signal recognition. Here, we validated the performance of MMR by comparing the recognition rates under different SNRs. From **Fig. 4b** of the main text, when the SNR was -20 dB, we have demonstrated that the recognition accuracies of patterns with and without MMR were about 99.5% and 34.0%, respectively. We constructed four different signals according to the signal components shown in **Supplementary Table 5**, and completed verification experiments at -25 dB, -30 dB, -35 dB and -40 dB, respectively. The comparison of recognition accuracy under different SNRs with and without MMR is shown in **Supplementary Fig. 16**. From the results, we found that the recognition accuracy with MMR decreases as the SNR decreases. Specifically, when the SNRs are -20 dB and -40 dB, the recognition accuracies with MMR are 99.5 % and 58.0%, respectively. This is because the signal components that can be measured by MMR decrease as the SNR decreases. From the results without MMR, we found that the recognition accuracy without MMR fluctuates below 40% as the SNR decreases. This is because the conventional piezoelectric mechanoreceptor cannot measure clear signal components since the signal with SNR below -20 dB cannot reach the detection limit.

We further demonstrated the improvement of recognition accuracy by increasing training samples. As shown in **Supplementary Fig. 17a**, when the SNR is -25 dB and the number of training samples is 200, the recognition accuracy with MMR is 92.5 %. Under the same conditions, we further increased the number of training samples from 200 to 400, and found that the recognition accuracy of test data can be increased from 92.5% to 99.5% as shown in **Supplementary Fig. 17b**. Therefore, we demonstrated that the recognition accuracy of testing data can be improved by increasing the training samples in the constructed intelligent sensing system.

Supplementary Note 14. Details of remote-vibration monitoring

For the demonstrated remote-vibration monitoring, we showed a remote-vibration scene of a piano playing, where we identified the piano playing inside the room by monitoring the vibrations on the exterior wall. Here, we presented an excerpt from the piano staves of the selected music (Mariage D'amour) as shown in **Supplementary Fig. 18**. From the measured results with MMR, the real-time spectrogram of musical scales could be monitored and obtained successfully as shown in **Supplementary Fig. 18a**. The measured signal with MMR presented clear time-frequency components that contained the real-time scale information of piano playing. In contrast, as shown in **Supplementary Fig. 18b**, the measured results without MMR presented unclear time-frequency components. This result showed that the vibrations of the exterior wall caused by the piano playing were extremely weak with strong background noise. In this demonstrated remote-vibration sensing, our designed MMR showed great potential for monitoring the weak vibrations that were difficult to be measured without MMR.

To further quantify the sensing performance of MMR in the demonstrated remote-vibration monitoring, we combined MMR and a deep neural network to realize the recognition of musical scales. Here, we carried out identification experiments of 14 musical scales, including 7 Do-Ti scales and 7 higher Do'-Ti' scales. Each scale has a vibration signal component of a specific frequency, which can be extracted by MMR and further input to the deep neural network for training and recognition. In this experiment, we constructed 1400 signal samples, 50% of which were used for training and the other 50% for recognition. We presented the recognition details as shown in **Supplementary Fig. 19**. From the recognition results with MMR, only two samples were identified incorrectly, and the overall recognition accuracy was 99.7% as shown in **Supplementary Fig. 19a**. In contrast, from the recognition results without MMR, most samples were identified incorrectly and the average recognition accuracy was 18.5%, which was far lower than the recognition performance with MMR as shown in **Supplementary Fig. 19b**. Therefore, MMR exhibited excellent detection advantages for remote-vibration monitoring[32], which may also have potential prospects for remote information acquisition and decryption in the field of security and confidentiality[19].

Supplementary Note 15. Details of the smart-driving assistance

For the demonstrated smart-driving assistance, we combined MMR and the artificial deep neural network to construct one in-vehicle intelligent sensing system as shown in **Supplementary Fig. 20a**, which can complete signal collection, information processing and decision execution. In this intelligent sensing system, the vehicle can autonomously report two running statuses including "Normal" and "Fault" to ensure driving safety. In this application scenario, we conducted experimental demonstrations with six engine vibration signals, including one normal signal and five faulty signals. The five fault signals come from the balance shaft on the intake side (Fault #1), the abnormal noise during acceleration (Fault #2), the camshaft on the intake side (Fault #3), the abnormal noise during the low-temperature start (Fault #4), and the four-position three-way valve

on the intake side (Fault #5), respectively. We presented the measured time-frequency representations of six signals with and without MMR as shown in **Supplementary Fig. 20b-g**. From the time-frequency representation results, we can find that the measured results with MMR presented clearer time-frequency components than that without MMR.

The noise during the vehicle movement is mainly divided into two categories: one is the external sound noise in the cab, and the other is the internal background noise caused by the attenuation of the micro-motion signal by the vibration transmission path. Since the MMR has no related impedance matching design, most of the noise in the cab is reflected by the MMR and cannot be transmitted into it. Thus, the external sound noise in the cab interferes little with the operation of MMR. Here we mainly focus on the internal background noise caused by the attenuation of the micro-motion signal in the transmission process. As shown in the Fig. 4e of the main text in the revised manuscript, we identified the health status of engine by monitoring the micro-motions on the cab platform far away from the engine. Early abnormal vibration signal from the engine is attenuated during transmission, leading to low signal-to-noise ratio and great difficulty to be detected. For the working mechanism of MMR, there are two processes to improve the signal-to-noise ratio: one is the mechanics-guide design with zero effective mass that enhances the time-varying frequency components of the micro-motions, and the other is the adaptive filtering algorithm in the computational multi-channel demodulation that reduces the background noise.

We further input the time-frequency representations with and without MMR into the constructed deep neural network, respectively. Here, we constructed 600 signal samples, 50% of which were used for training and the other 50% for recognition. We presented the recognition details as shown in **Supplementary Fig. 21**. From the recognition results with MMR, 14 samples were identified incorrectly, and the overall identification accuracy was 95.3% shown in **Supplementary Fig. 21a**. In contrast, from the recognition results without MMR, most samples were identified incorrectly and the average identification accuracy rate was 14.3%, which was far lower than the recognition performance with MMR as shown in **Supplementary Fig. 21b**. The high recognition accuracy in the demonstrated smart-driving assistance was attributed to the highly sensitive and broadband property of MMR. Therefore, MMR showed great potential in smart-driving assistance, which was expected to play an important role in the Internet of Vehicles[33] and the Internet of Things[34].

Supplementary Note 16. Details of structural health monitoring

To further confirm the working ability of MMR and explore the possibility of application in industrial scenario, we added a demonstration of structural health monitoring as shown in **Supplementary Fig. 22**. Rotating machinery is the main body of mechanical equipment in aerospace and other industrial fields, and the research on fault diagnosis of rotating machinery is of great significance for the stable operation of equipment. **Supplementary Fig. 22a** shows a twin-engine multi-purpose helicopter which is a typical representative of rotating machinery in aerospace. Its transmission system includes main rotor shaft, main reducer, tail transmission shaft,

connecting shaft and tail reducer as shown in **Supplementary Fig. 22b**. The tail transmission shaft is a slender shaft located between the main reducer and the tail reducer, on which four bearings of identical type are arranged as support and transmission. Due to the limitation of sensor measurement arrangement location, identifying weak faults of remote bearings is crucial to its structural health monitoring.

For the helicopter transmission system, we built a rotor test bench for structural health monitoring of slender tail shaft, which is a more complex application environment due to the variety and unpredictability of early bearing failures. As shown in **Supplementary Fig. 22c**, we arrange MMR on the top of the right end bearing seat, and then replace the bearing inside the left end seat housing with an early weak failure bearing. As shown in **Supplementary Fig. 22d**, the remote bearing failure is sensed by the MMR and reconstructed to obtain the signal $s(t)$, which is further combined with the deep neural network (DNN) to realize the Monitoring and identification of early weak faults. As shown in **Supplementary Fig. 23**, we present the signal measurement details of early weak faults. We first machined three bearings with early failure, including inner race faults (Fault #1), outer race faults (Fault #2) and rolling element faults (Fault #3). Next, experiments were carried out under the normal state (Normal) and three faulty states, respectively. It can be seen that the test results based on MMR show clear time-frequency representations relatively, and the time-varying frequency components corresponding to each faulty state are different. In contrast, the four signals measured by the conventional piezoelectric accelerometer under the normal state and three fault states are similar. The results show that the early faults generated by the left end bearing are greatly attenuated when transmitted to the right end bearing seat, which is difficult for the conventional piezoelectric accelerometer to measure.

Then, we input the time-frequency representations with and without MMR into the constructed deep neural network, respectively. Here, we constructed 400 signal samples, 50% of which were used for training and the other 50% for recognition. We presented the recognition details as shown in **Supplementary Fig. 24**. From the recognition results with MMR, the overall identification accuracy was 96% shown in **Supplementary Fig. 24**a. In contrast, from the recognition results without MMR, most samples were identified incorrectly and the average identification accuracy rate was 27%, which was far lower than the recognition performance with MMR as shown in **Supplementary Fig. 24**b. The high recognition accuracy in the demonstrated smart-driving assistance was attributed to the highly sensitive and broadband property of MMR.

Supplementary Note 17. Details of running status detection of rotor test bench

In practical applications, the vibration and micro-motions during MMR detection can be complex. As shown in **Supplementary Figs. 25a** and **25b**, we mainly divide the direction of micro-motion into two types: one is z-direction stimulation, and the other is non-z-direction stimulation. For the z-direction stimulation shown in **Supplementary Fig. 25a**, the direction of micro-motion stimulation is consistent with the d_{33} detection direction of MMR, which is the most beneficial detection condition to maximize the transmission of micro-motion into MMR. For the non-z-

direction stimulation shown in **Supplementary Fig. 25b**, the direction of the micro-motion stimulation has a certain angle with the z-direction. In this case, we can decompose the micro-motion S into the z-direction and the parallel direction to obtain S_z and S_o, respectively. Since MMR is mono responded in z-direction, the S_z component can be enhanced with the local resonance as shown in **Supplementary Fig. 25c**, while S_o component cannot be detected. In practical scenes, the micro-motion feature components may be different in different directions. If the S_z contains sufficient signal feature components, we can still use MMR to detect the micro-motion signals.

As shown in **Supplementary Fig. 25d**, we demonstrate the use of MMR to detect the movement state of a rotor test bench during the start-up process. The architecture of the whole rotor test bench is shown in **Supplementary Fig. 22**. We mounted the MMR above the bearing block on the right end. Then we started the test bench from 0 RPM to 4000 RPM, and kept it running stably at 4000 RPM. During start-up, the vibration generated by the rotor was time-varying and multi-directional, which caused the z-direction vibration to be transmitted to the MMR through the bearing block. For MMR, the vibration source was extremely complex, and only the corresponding vibration component in the z direction can be detected. The time-domain and time-frequency presentation (unified) of measured signals during the start-up process were shown in **Supplementary Figs. 25e** and **25f**, from which we found that MMR detected three operating movement states: The first state was a stationary state from 0 to 5 s, during which the rotor test rig was ready to start and there was no signal component; the second state was a speed up state from 5 to 35 s, during which the rotor test bench accelerated from 0 to 4000 RPM and the frequency of signal component moved to high frequency gradually; the third state was the steady state after 35 s, during which the rotor test bench ran stably at 4000 RPM and the signal component was in a stable state.

In summary, we provide a demonstration that MMR could detect three movement states of the rotor test bench during the start-up process. In practical applications, the vibration and micro-motions during MMR detection, especially in directionality, can be complex. We demonstrate that the realization of micro-motion sensing by MMR needs to meet the following two conditions: one is that the micro-motion has a micro-motion signal component in the corresponding z direction (d_{33} direction of MMR); the other is that the micro-motion signal component reaches the detection limit of MMR and is within the frequency range.

Supplementary Note 18. Details of stability and reliability in different application scenarios

Moreover, we also gave specific experimental data (with normalized amplitude) in demonstrated remote-vibration monitoring, smart-driving assistance and structural health monitoring as shown in **Supplementary Fig. 26.** From the results as shown in **Supplementary Fig. 26a**, we gave the specific data of 7 Do-Ti scales and 7 higher Do'-Ti' scales in the demonstrated remote-vibration monitoring scenario. Each of the 14 scales lasts 0.5 seconds and is cycled twice. We then calculated the zero drift of the musical scale signal as shown in **Supplementary Fig. 26b**, from which we found that the magnitude of the zero drift is less than 0.01. More, we also gave the specific

experimental data of failed engine signal in the demonstrated smart-driving assistance. As shown in **Supplementary Fig. 26c** and **Supplementary Fig. 26d**, we calculated that the zero drift of the normal engine signal is less than 0.01. Similarly, the zero drift of the failed bearing signal in the demonstrated structural health monitoring is also less than 0.01 as shown in **Supplementary Fig. 26e** and **Supplementary Fig. 26f**. In summary, we gave specific experimental data in demonstrated remote-vibration monitoring, smart-driving assistance and structural health monitoring, which demonstrated that MMR showed strong stability and reliability in the measurement of amplitude stability in different application scenarios.

Supplementary Note 19. Integrated sensing of MMR

In our experiment, a data acquisition equipment was used to collect the electrical signal generated by MMR, and the micro-motion signal was reconstructed through a computational multi-channel demodulation algorithm in the computer. As shown in **Supplementary Fig. 27**a, we also provided some exploration possibilities that integrates the central processing unit (CPU) and the MMR. Here the CPU integrates data acquisition, storage and calculation, which can be embedded in the MMR and connects the output of each unit cell to the corresponding acquisition channel as shown in **Supplementary Fig. 27**b. In this way, we can integrate MMR into an all-in-one sensing system as shown in **Supplementary Fig. 27**c, including sensing, storage and computing functions. This design can make the entire sensing system simpler and more portable.

Supplementary Figures

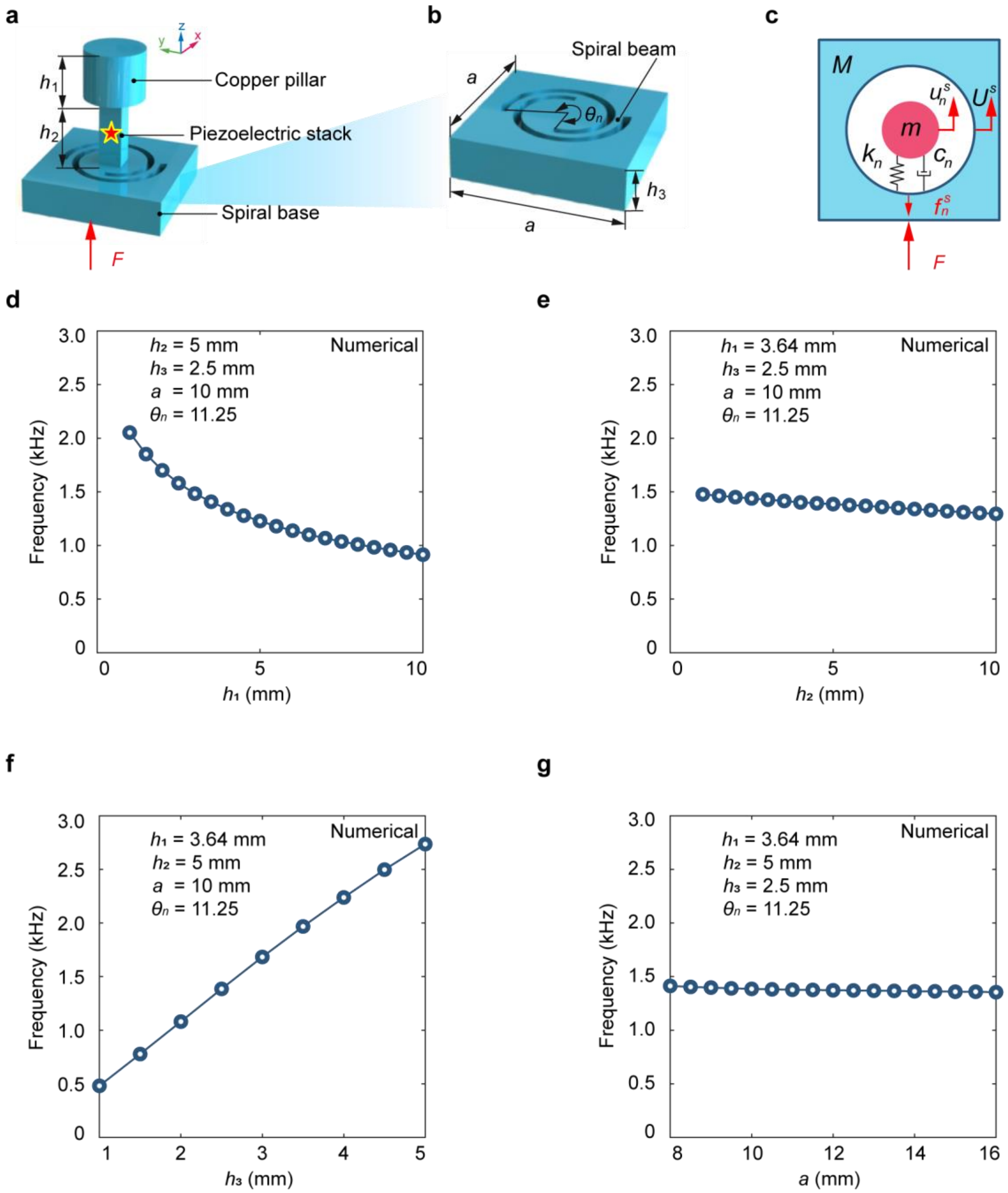

Supplementary Fig. 1 | Frequency-dependent effective piezoelectric coefficient and parameter study. a, Single unit cell with applied excitation F in the z-direction. **b**, Spiral base of the unit cell. **c**, The simplified dynamical model of the unit cell with frequency-dependent zero effective mass. **d**, The effect of h_1 on ω_0. **e**, The effect of h_2 on ω_0. **f**, The effect of h_3 on ω_0. **g**, The effect of a on ω_0.

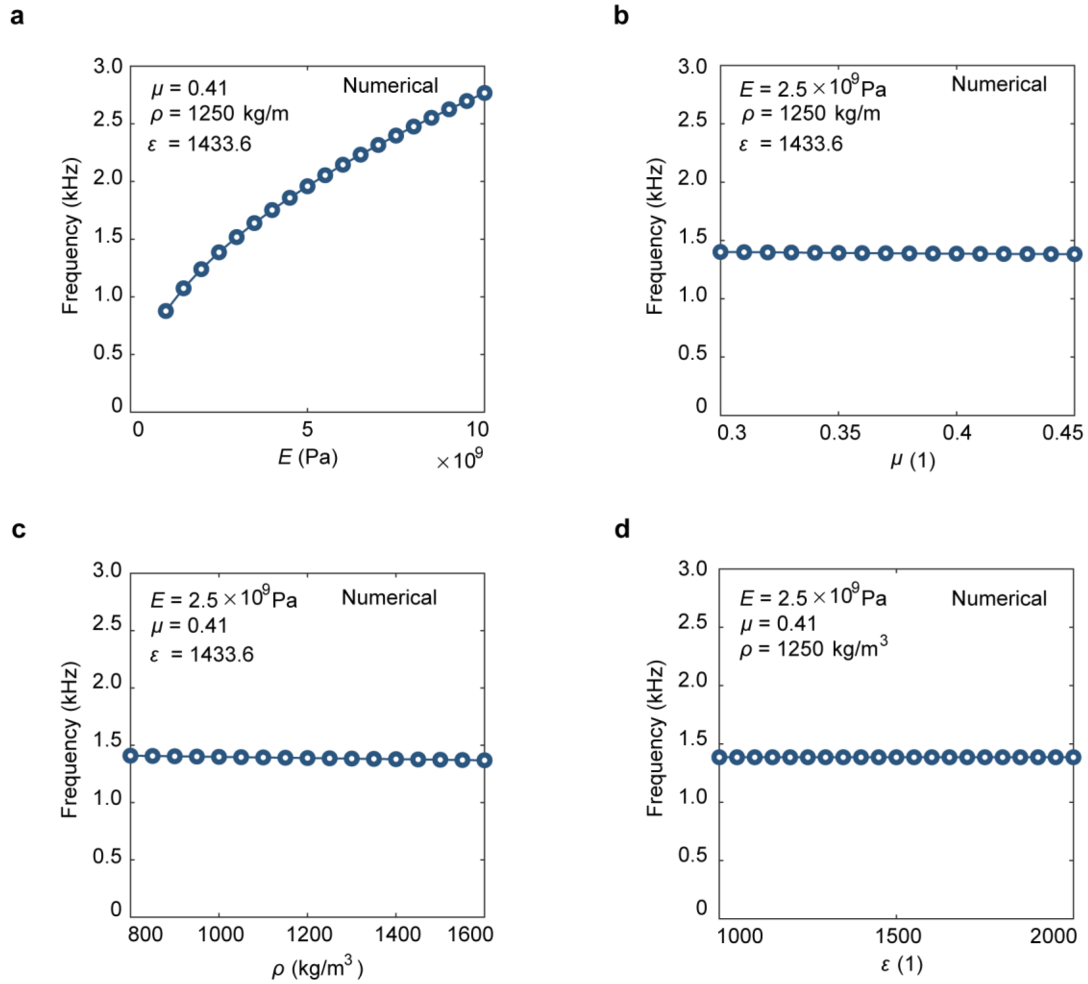

Supplementary Fig. 2 | Study on mechanical and physical properties. a, The effect of E on ω_0. **b**, The effect of μ on ω_0. **c**, The effect of ρ on ω_0. **d**, The effect of ε on ω_0.

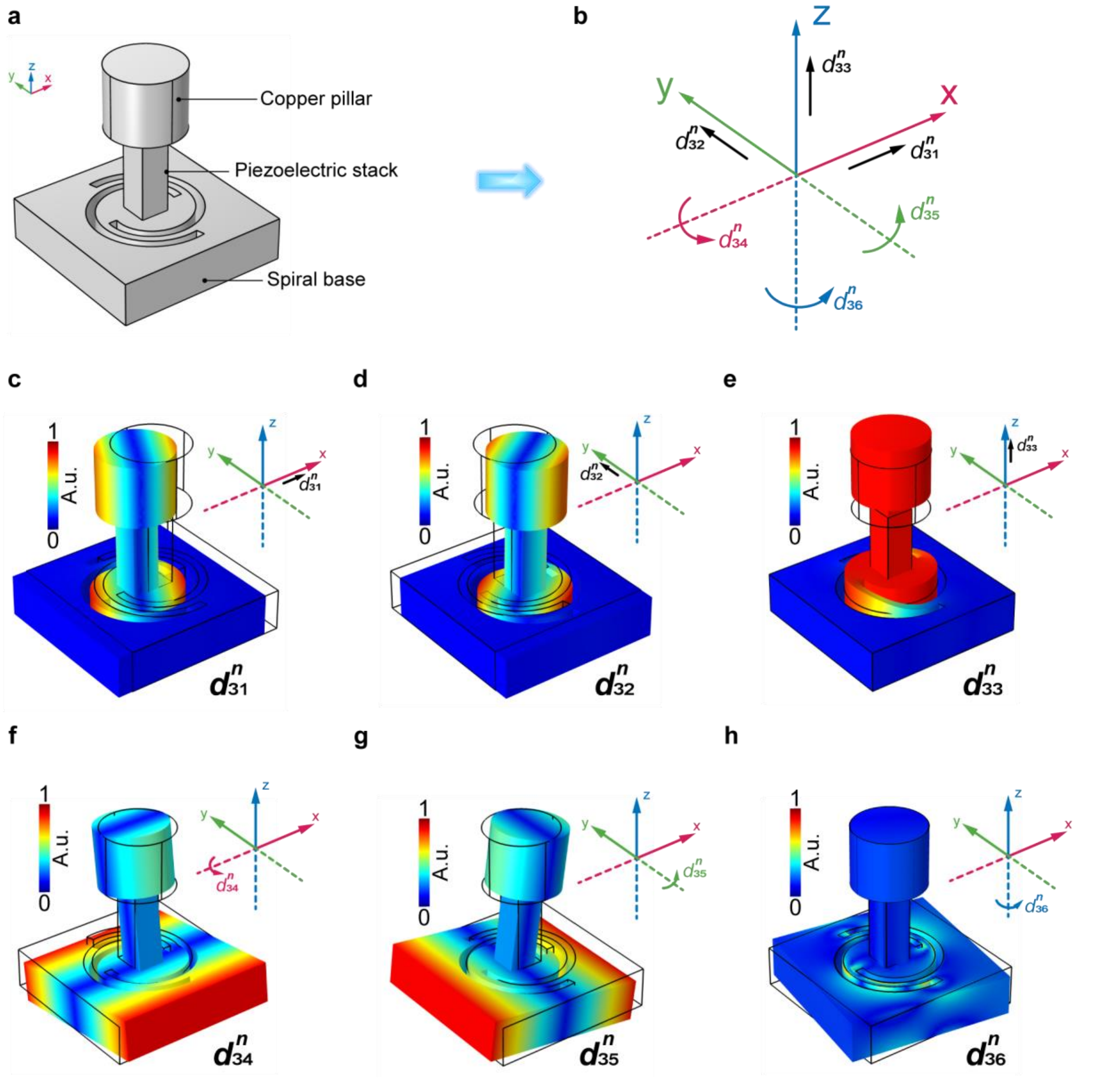

Supplementary Fig. 3 | Response simulation of unit cell under excitation in different directions. a, The unit cell model. **b**, Six possible directions of micro-motion excitation on the spiral base. **c**, Simulation in six different directions corresponding to (**c**) "d_{31}", (**d**) "d_{32}", (**e**) "d_{33}", (**f**) "d_{34}", (**g**) "d_{35}" and (**h**) "d_{36}".

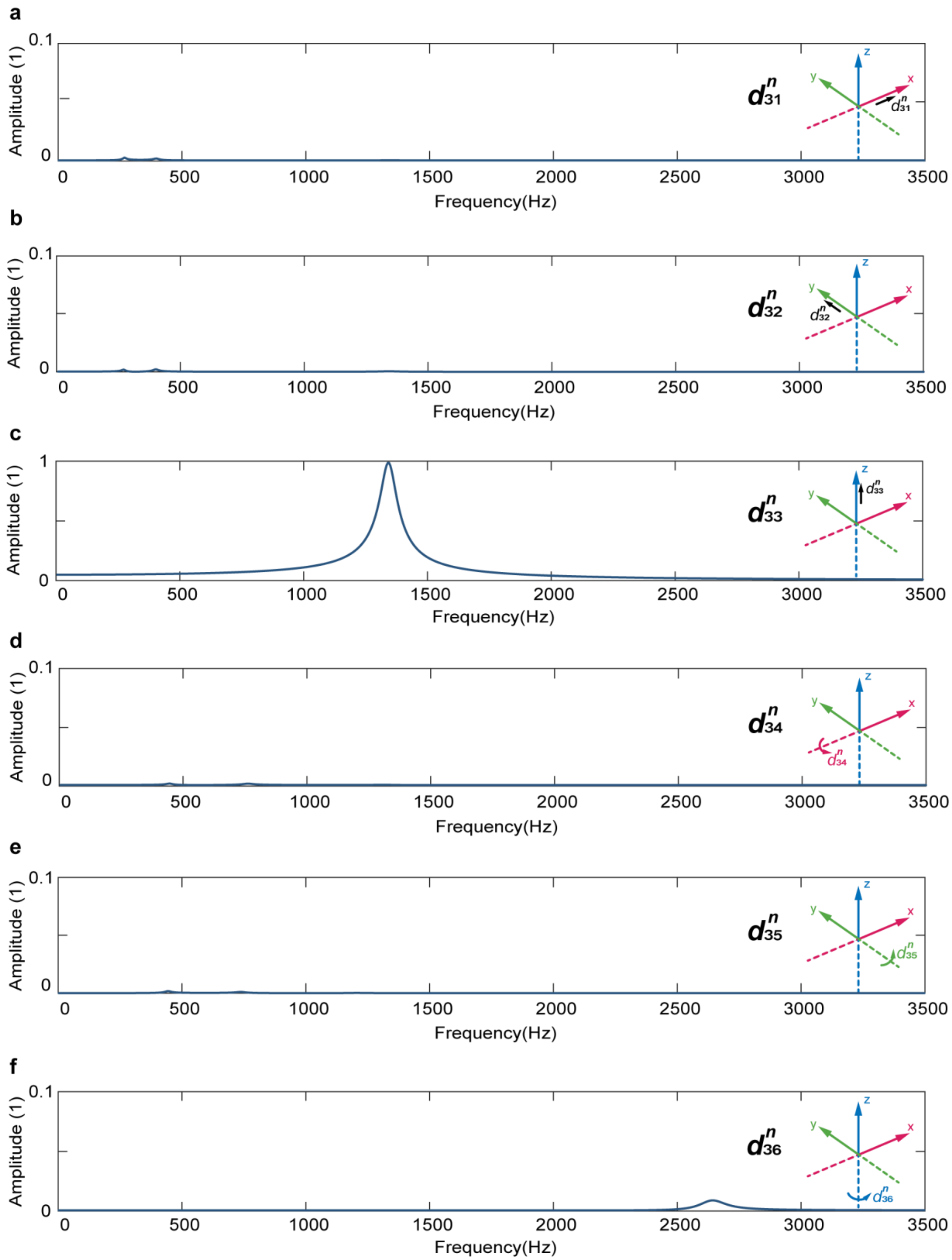

Supplementary Fig. 4 | Response details of unit cell in different directions. The voltage output amplitude of the unit cell in six directions corresponds to (**a**) "d_{31}", (**b**) "d_{32}", (**c**) "d_{33}", (**d**) "d_{34}", (**e**) "d_{35}" and (**f**) "d_{36}" under equal-amplitude swept-frequency micro-motion excitation.

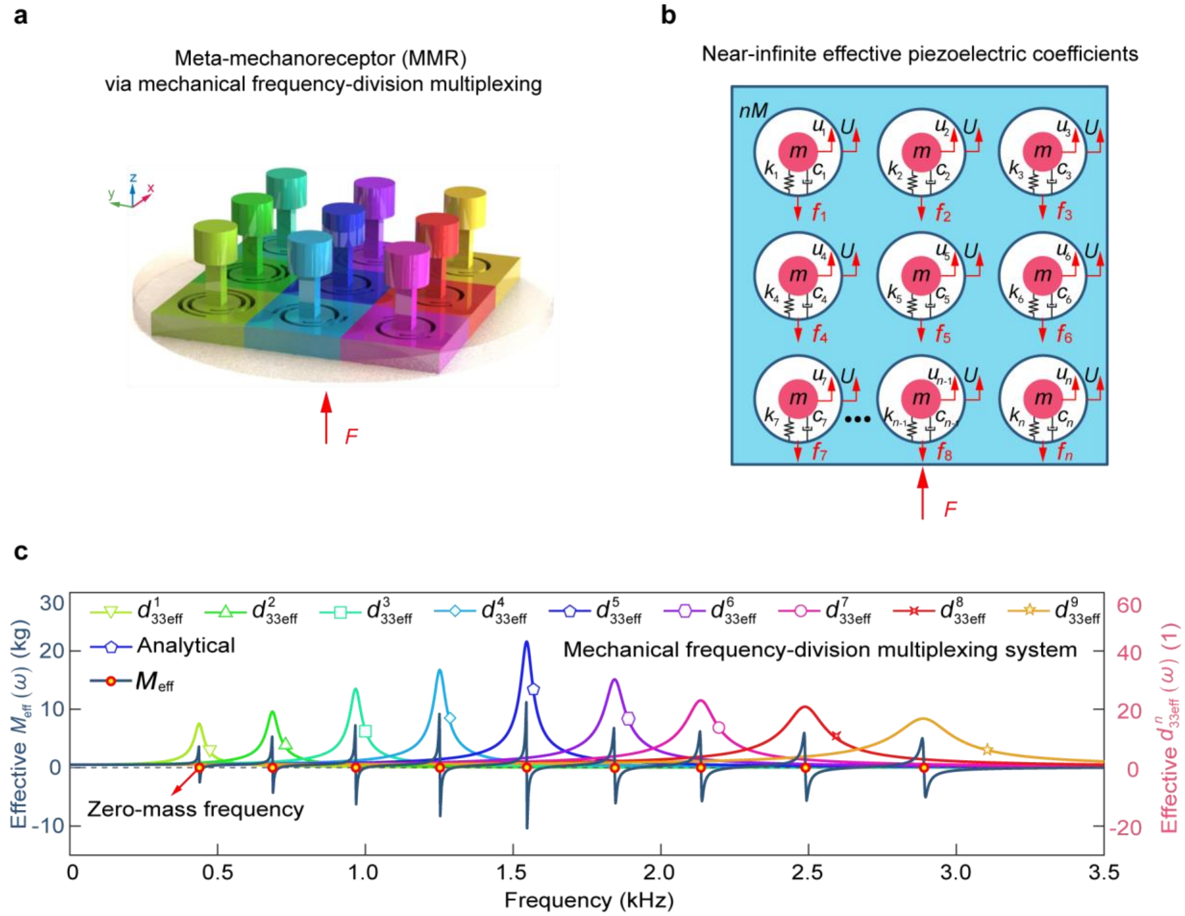

Supplementary Fig. 5 | Dynamical model of the mechanical frequency-division multiplexing system. a, MMR with applied excitation F in the z-direction. **b**, The global dynamical model of MMR. **c**, Distributed zero effective masses and frequency-dependent effective piezoelectric coefficients (normalized) with enhanced micro-motion sensing in the mechanical frequency-division multiplexing system.

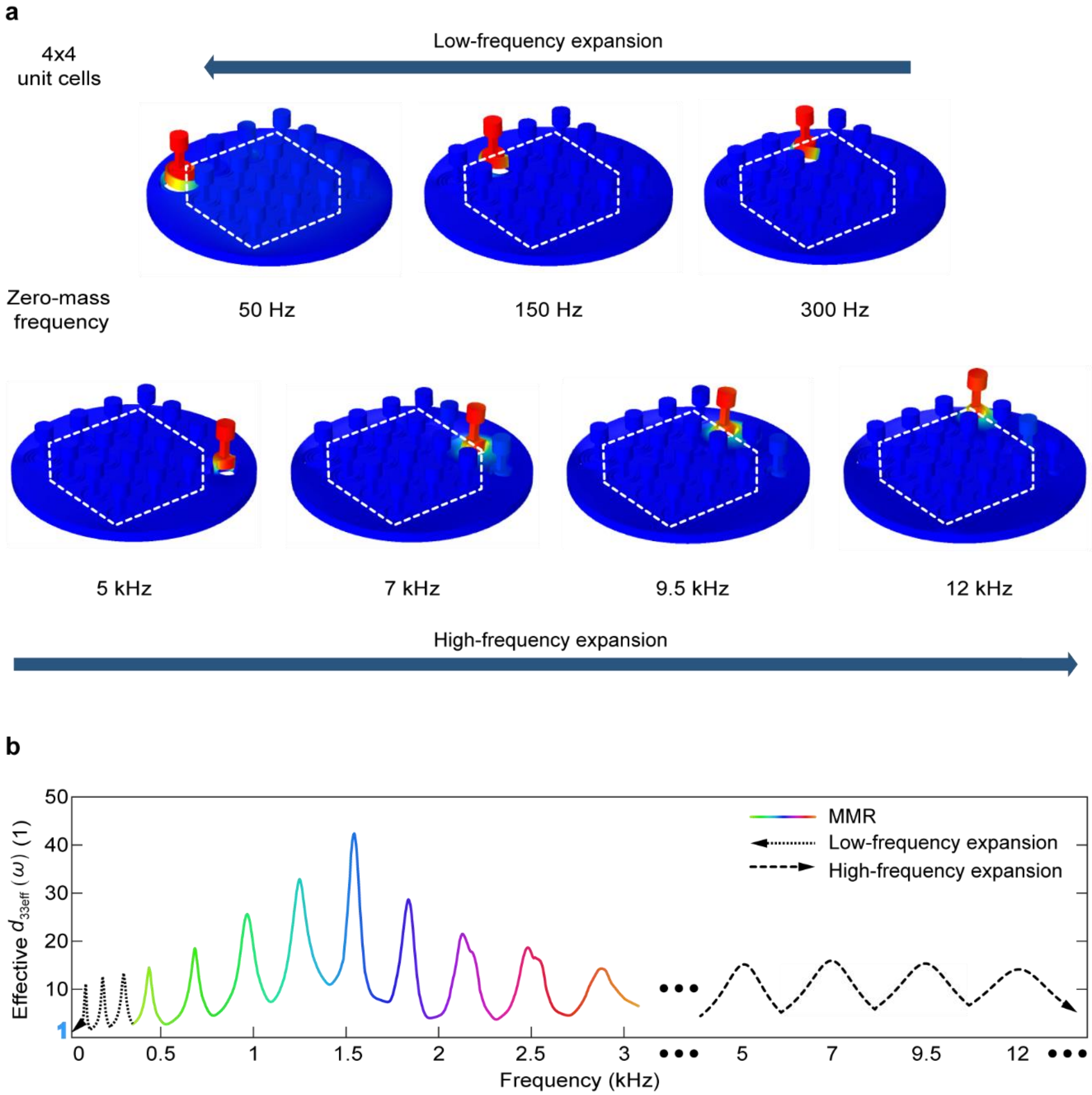

Supplementary Fig. 6 | Bidirectional expansion of working frequency band in MMR. a, The tailored design of zero-mass frequency by individually varying the θ_n of the unit cell. The extended 4×4 units based on the original 3×3 units denoted by the white box in MMR. **b**, The bidirectional expansion of the working frequency band in 0-12 kHz.

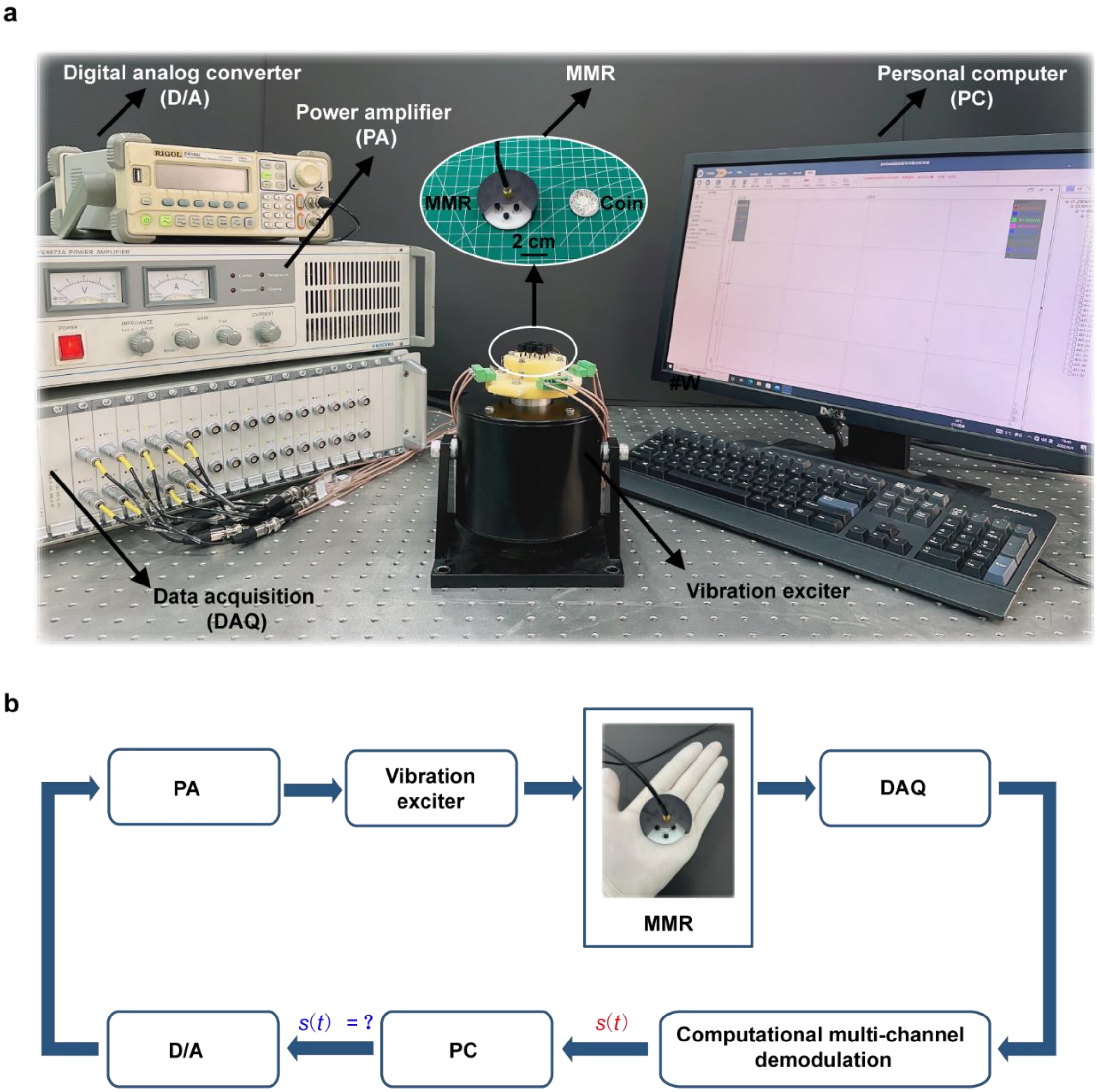

Supplementary Fig. 7 | Experiments for the performance verification of MMR. a, Photograph of the experimental setup. **b**, Schematic diagram of the experimental layout.

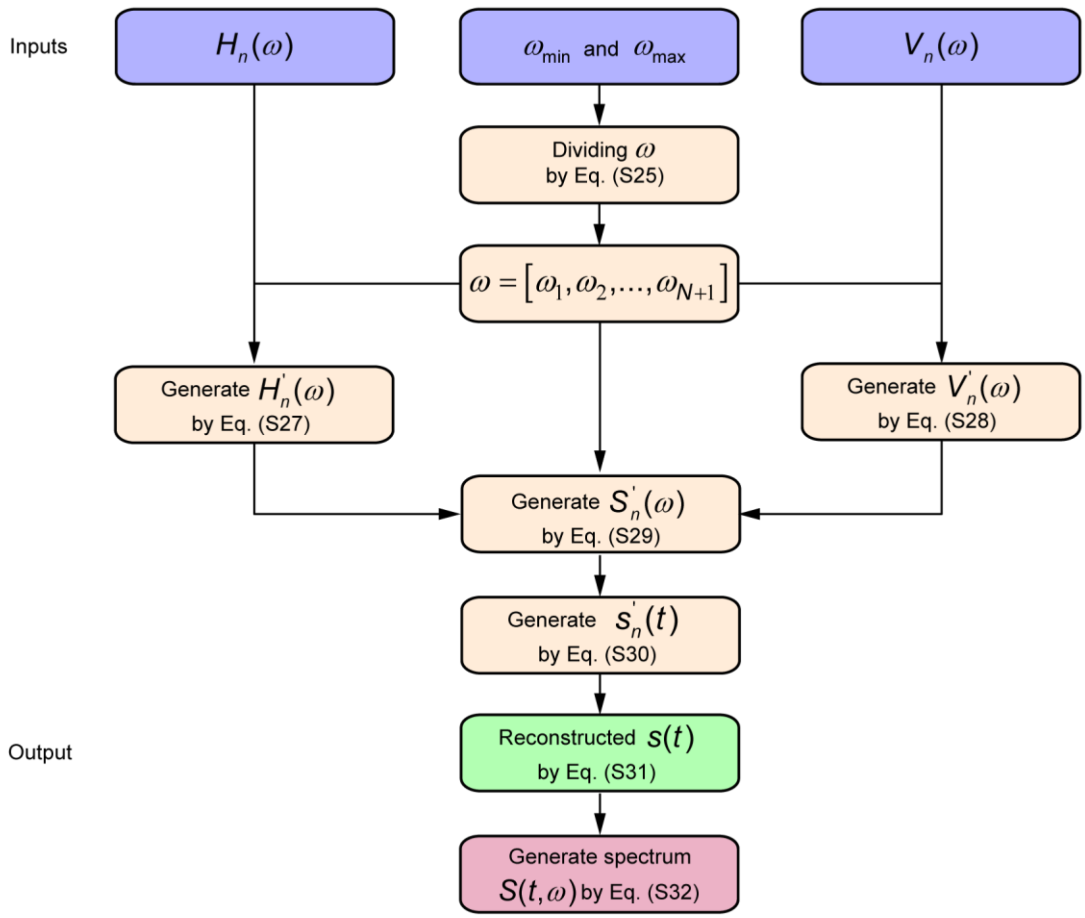

Supplementary Fig. 8 | Signal reconstruction with computational multi-channel demodulation. Blue blocks at the top represent the information inputs. Orange blocks in the middle are the information processes. Green block denotes the output time-domain signal. Purple block at the bottom is the time-frequency representation of the reconstructed signal.

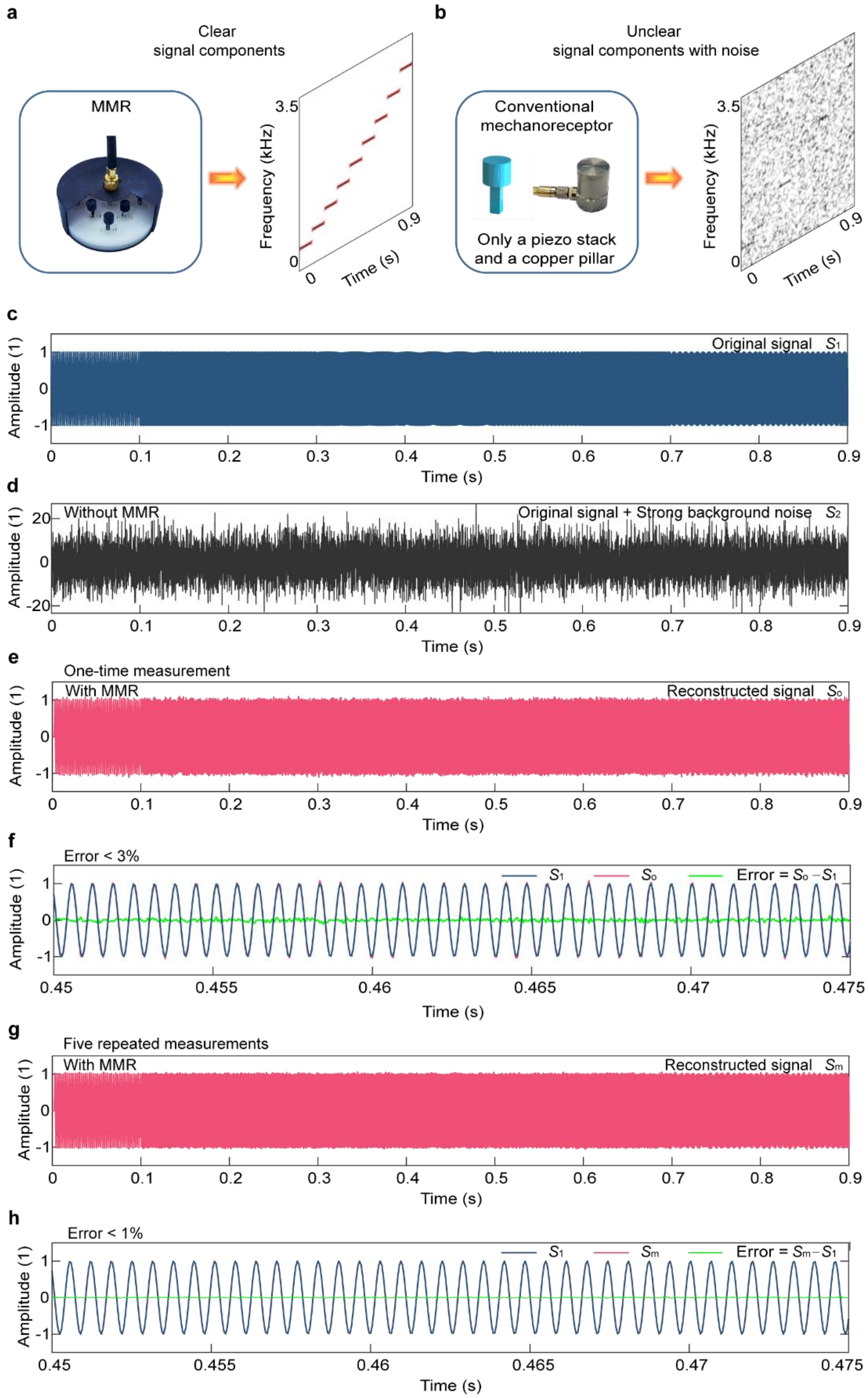

Supplementary Fig. 9 | Comparison between the results measured with and without MMR. **a**, The clear signal components with MMR. **b**, The unclear signal components without MMR. **c**, The constructed original signal S_1 consisting of nine harmonic components. **d**, At a signal-to-noise ratio (SNR) of -20 dB, the measured signal S_2 without MMR has strong background noise. **e**, One-time measurement S_o with MMR has (**f**) less than 3% error via computational multi-channel demodulation. **g**, Average of five repeated measurements S_m with MMR has (**h**) less than 1% error.

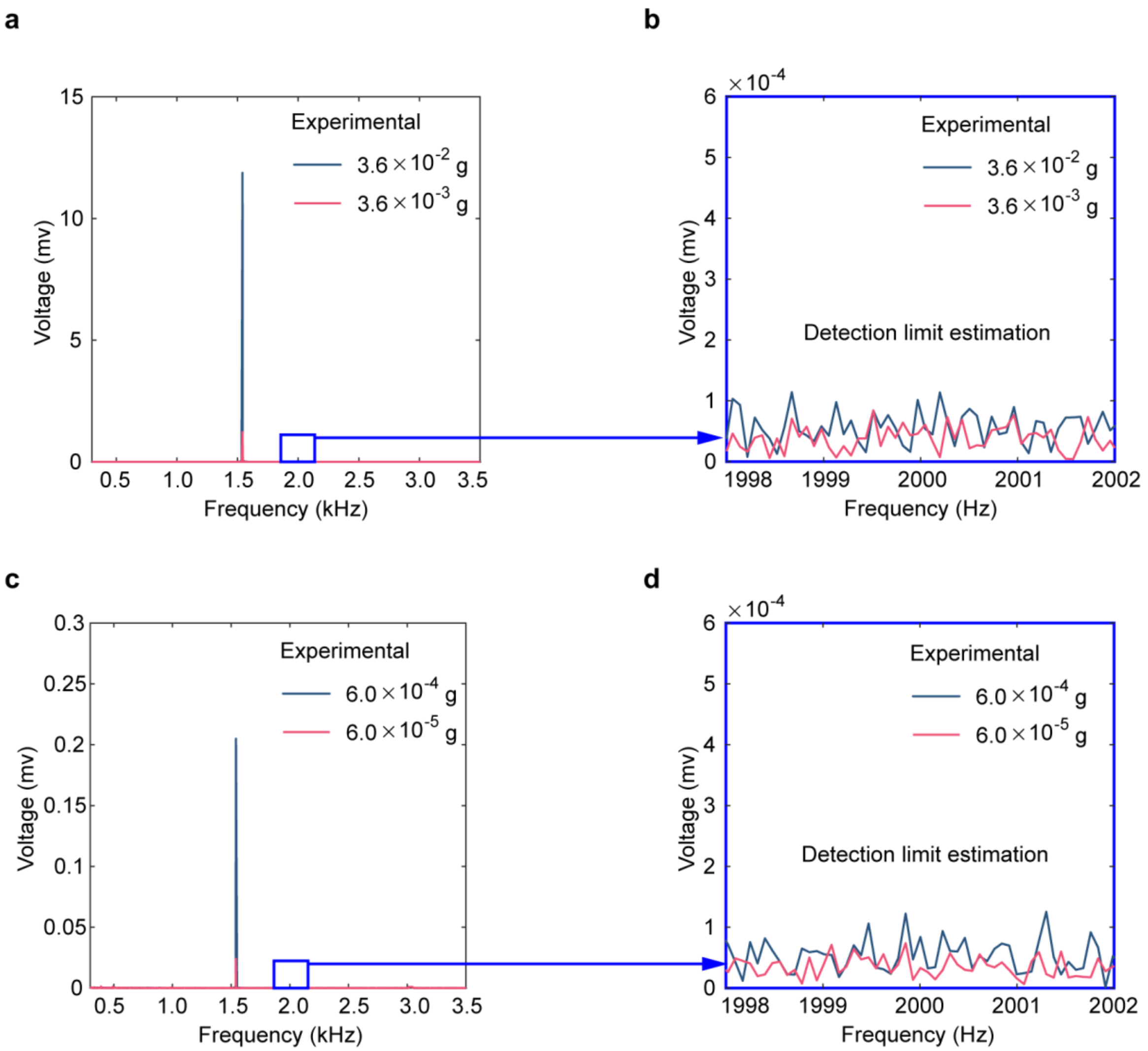

Supplementary Fig. 10 | Detection limit study. a, Frequency spectrum of MMR at 1540 Hz with a stimulation of 3.6×10^{-2} g and 3.6×10^{-3} g in the *z*-direction and (**b**) detection limit estimation. **c**, Frequency spectrum of MMR at 1540 Hz with a stimulation of 6.0×10^{-4} g and 6.0×10^{-5} g in the *z*-direction and (**d**) detection limit estimation.

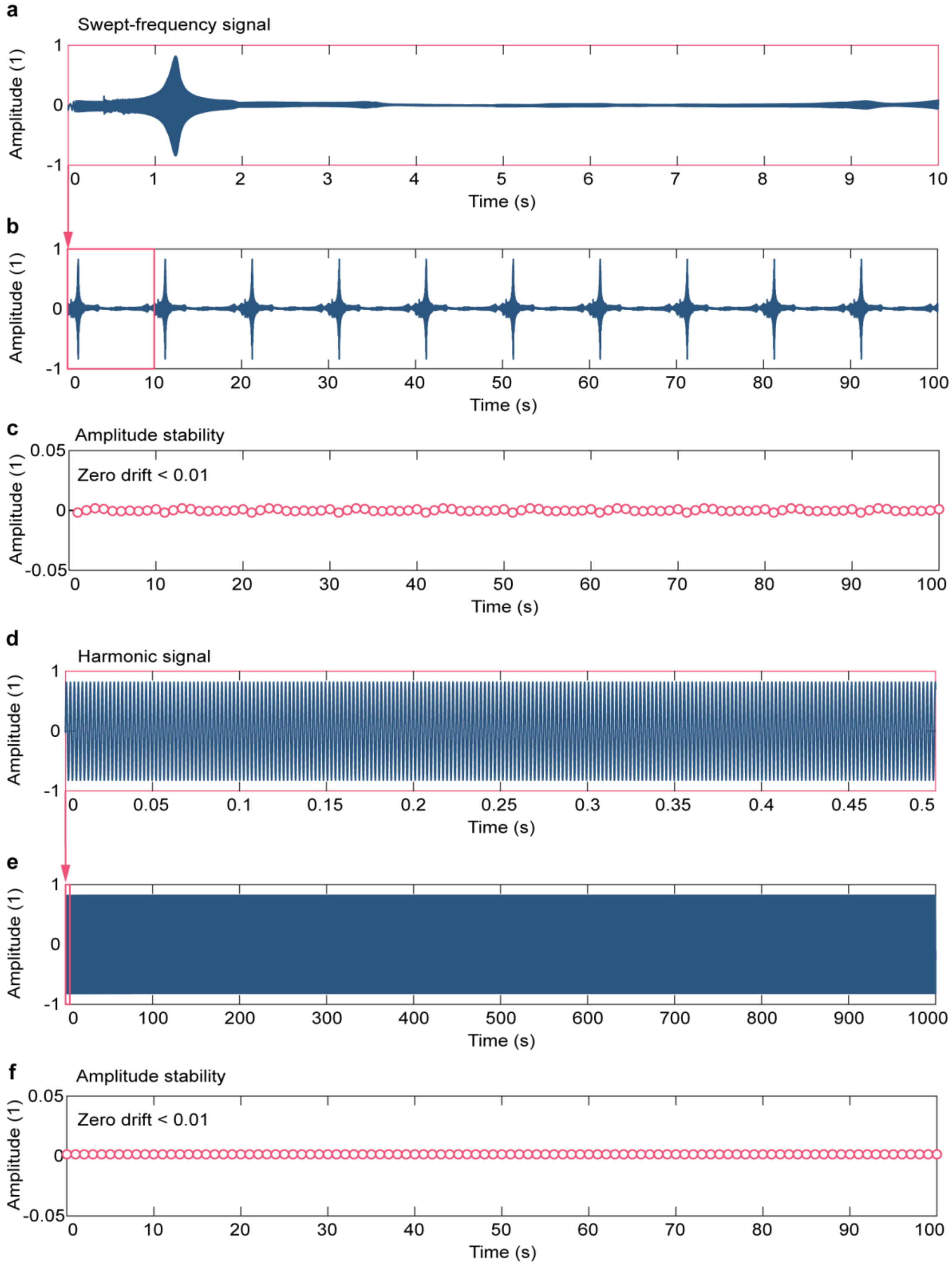

Supplementary Fig. 11 | Stability and reliability under long-time measurement. a, Swept-frequency signal under single cycle. **b**, Swept-frequency signal under ten cycles. **c**, Zero drift of measured amplitude under ten cycles. **d**, Measured amplitude of harmonic signal at 436.6 Hz. **e**, 1000-second continuous amplitude measurement of harmonic signals at 436.6 Hz. **f**, Zero drift of measured amplitude under continuous measurement of 1000 seconds.

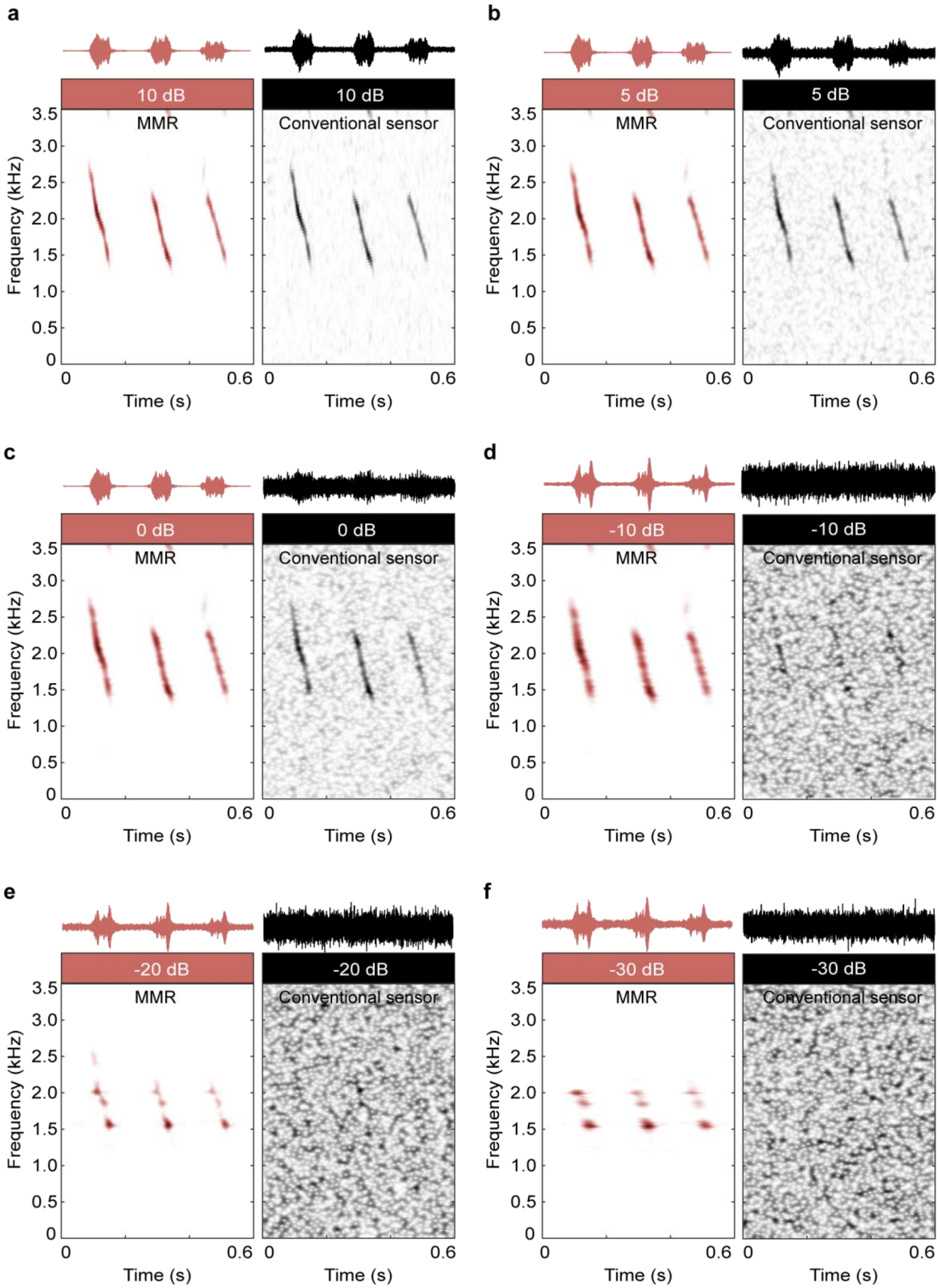

Supplementary Fig. 12 | Stability and reliability at different signal-to-noise ratios (SNRs). a, Micro-motion sensing performance at a SNR of 10 dB. **b**, Micro-motion sensing performance at a SNR of 5 dB. **c**, Micro-motion sensing performance at a SNR of 0 dB. **d**, Micro-motion sensing performance at a SNR of -10 dB. **e**, Micro-motion sensing performance at a SNR of -20 dB. **f**, Micro-motion sensing performance at a SNR of -30 dB.

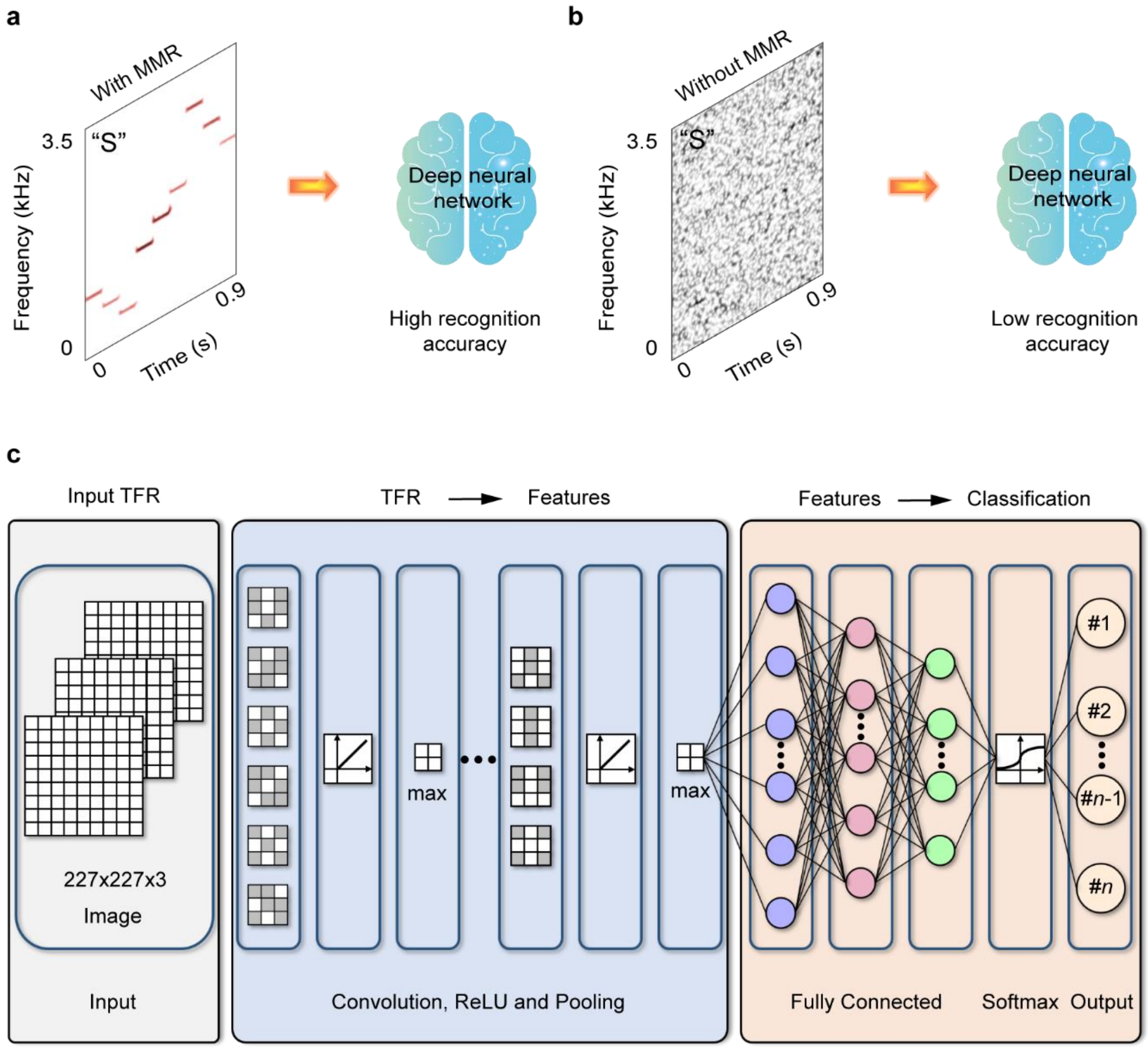

Supplementary Fig. 13 | Illustration of artificial deep neural network. a, The clear time-frequency representations by MMR are transported to a deep neural network to complete the training and recognition algorithms, which achieves a high recognition accuracy. **b**, The unclear time-frequency representations without MMR lead to a low recognition accuracy. **c**, Schematic diagram of the employed deep neural network. The time-frequency representation was briefly represented as TFR.

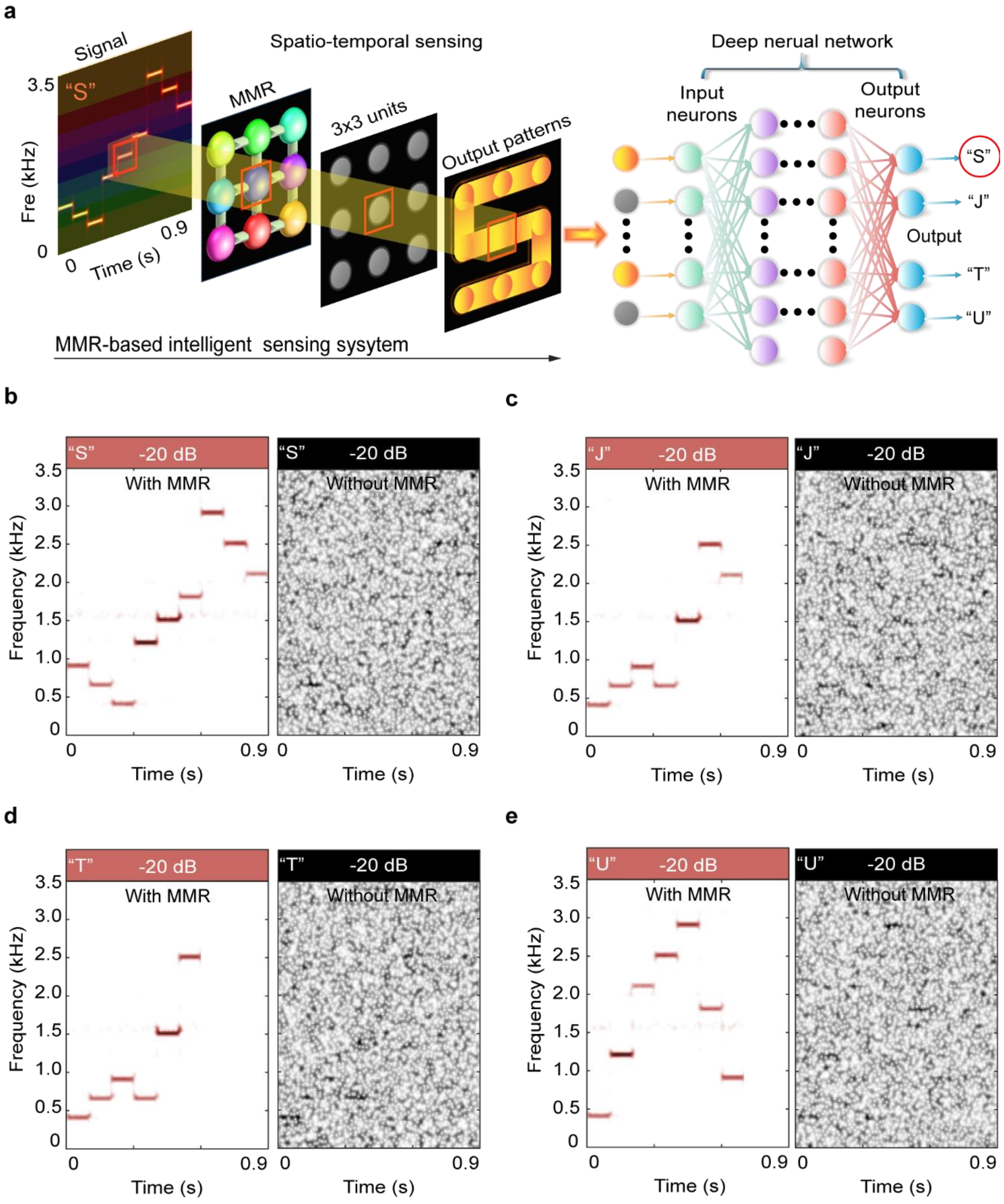

Supplementary Fig. 14 | Details of signal measurements in spatio-temporal sensing. a, Schematic of spatio-temporal sensing via MMR-based intelligent sensing system. The measured signals corresponding to (**b**) "S", (**c**) "J", (**d**) "T" and (**e**) "U" with and without MMR at the SNR of -20 dB. Here, "SJTU" is the abbreviation of Shanghai Jiao Tong University.

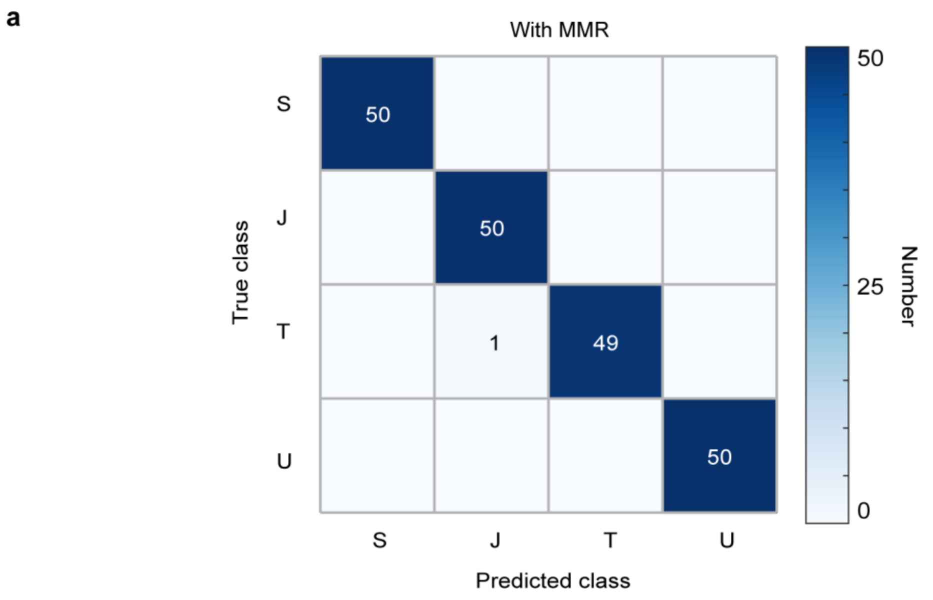

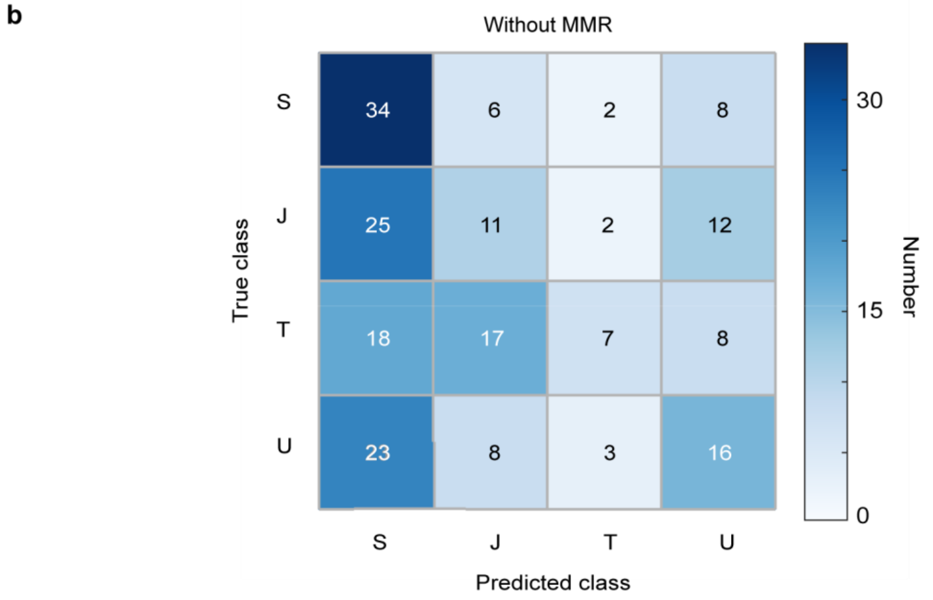

Supplementary Fig. 15 | Recognition comparison with and without MMR in spatio-temporal sensing. a, The average recognition accuracy with MMR is 99.5%. **b**, The average recognition accuracy without MMR is 34.0%.

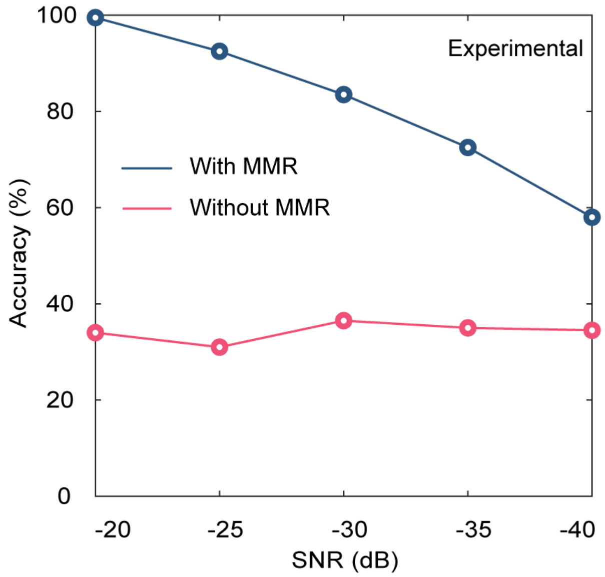

Supplementary Fig. 16 | Comparison of recognition accuracy under different SNRs with and without MMR.

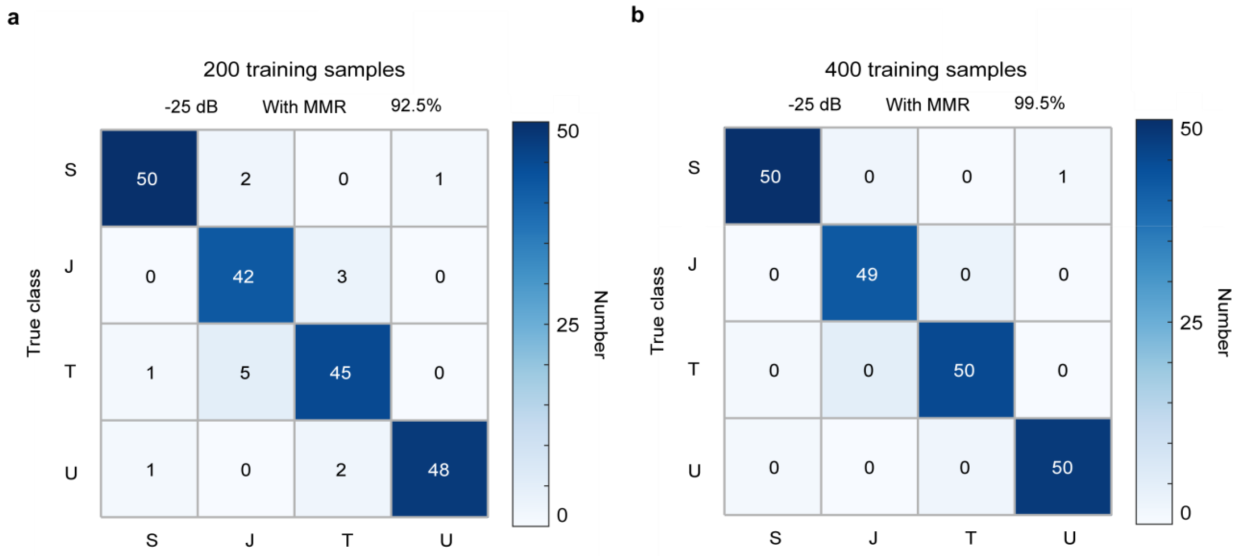

Supplementary Fig. 17 | Improvement of recognition accuracy by increasing training samples. The recognition accuracy corresponding to (**a**) 200 and (**b**) 400 training samples under -25 dB.

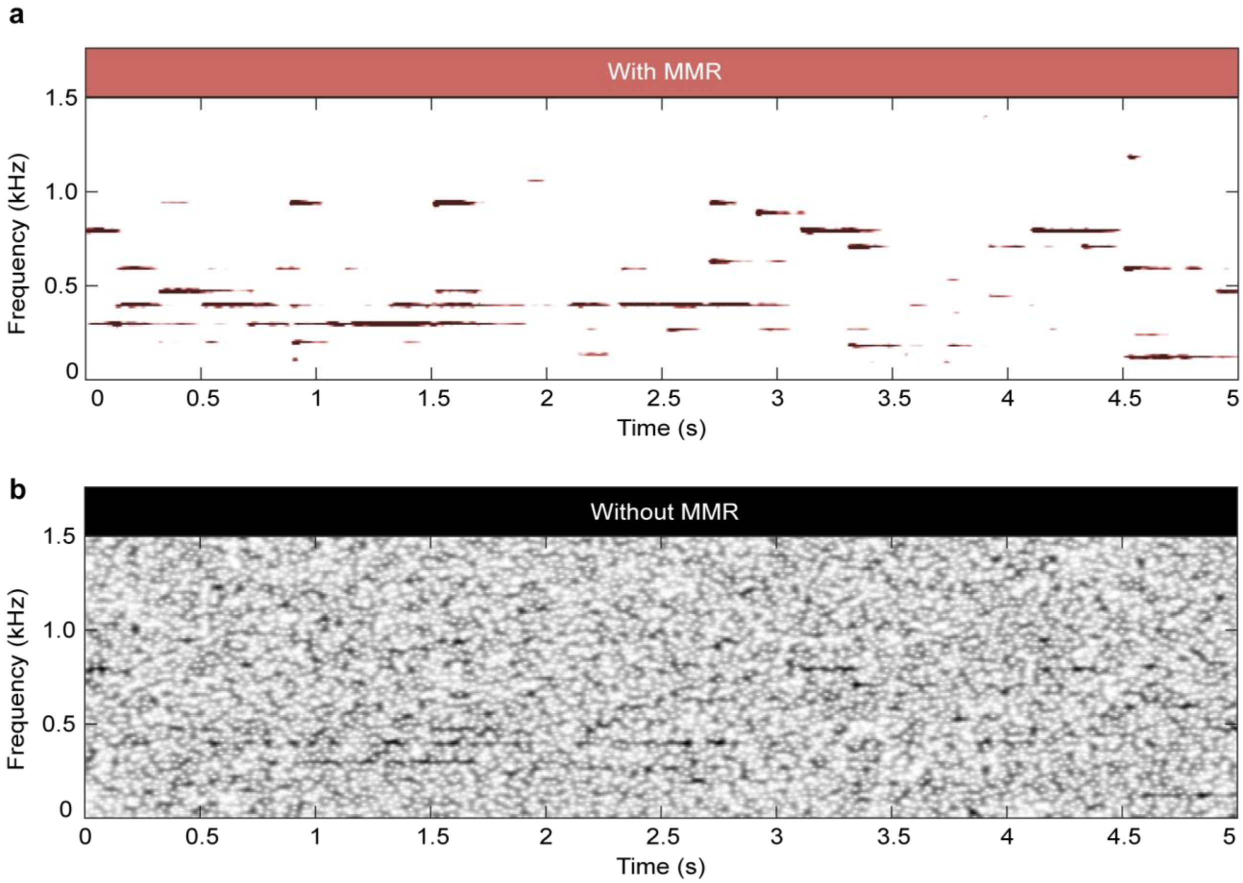

Supplementary Fig. 18 | Details of signal measurements in remote-vibration monitoring. a, The measured signal with MMR has a clear real-time spectrogram of musical scales. **b**, The measured signal without MMR has strong background noise.

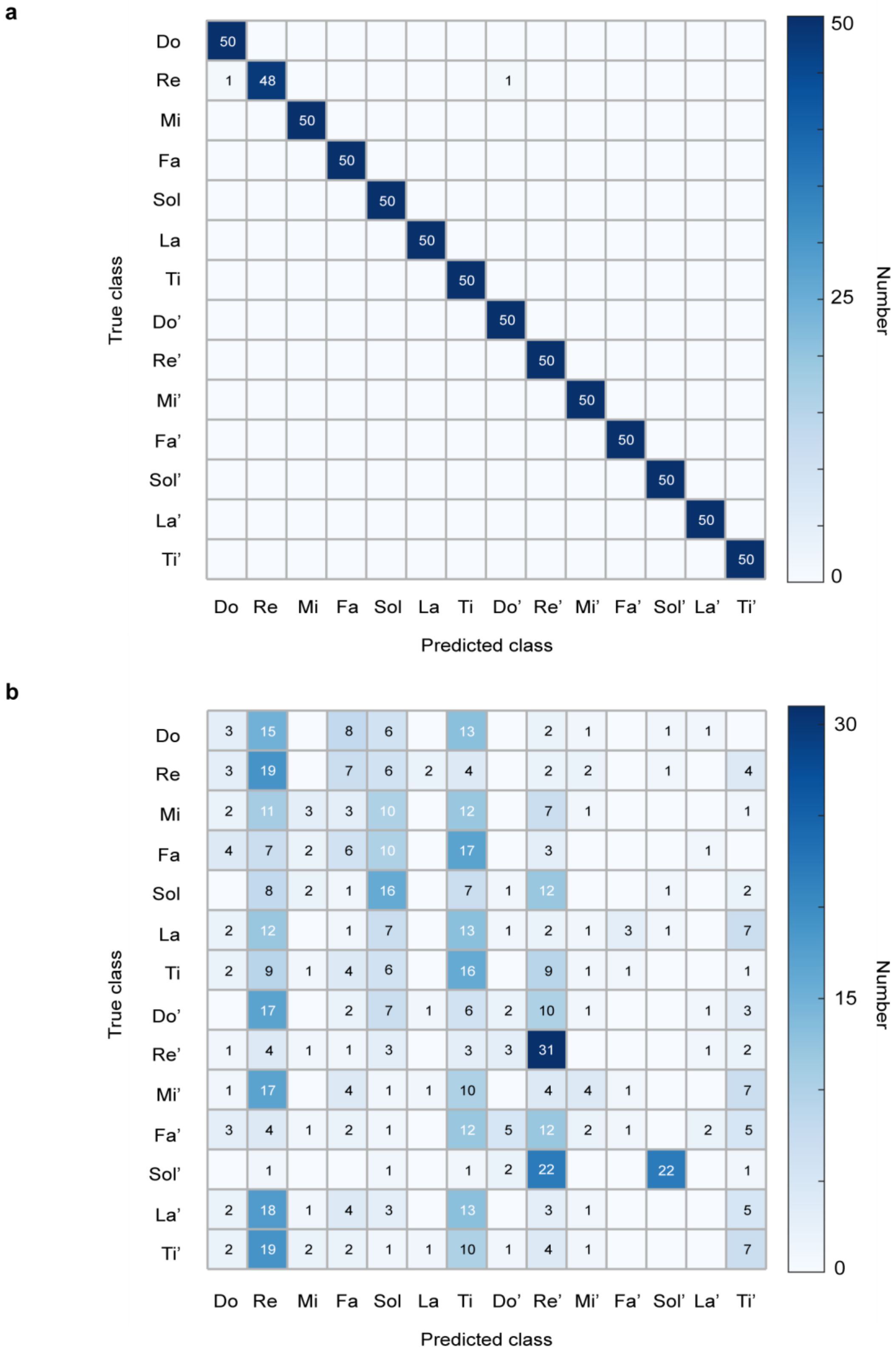

Supplementary Fig. 19 | Recognition comparison with and without MMR in remote-vibration monitoring. a, The average recognition accuracy with MMR is 99.7%. **b**, The average recognition accuracy without MMR is 18.5%.

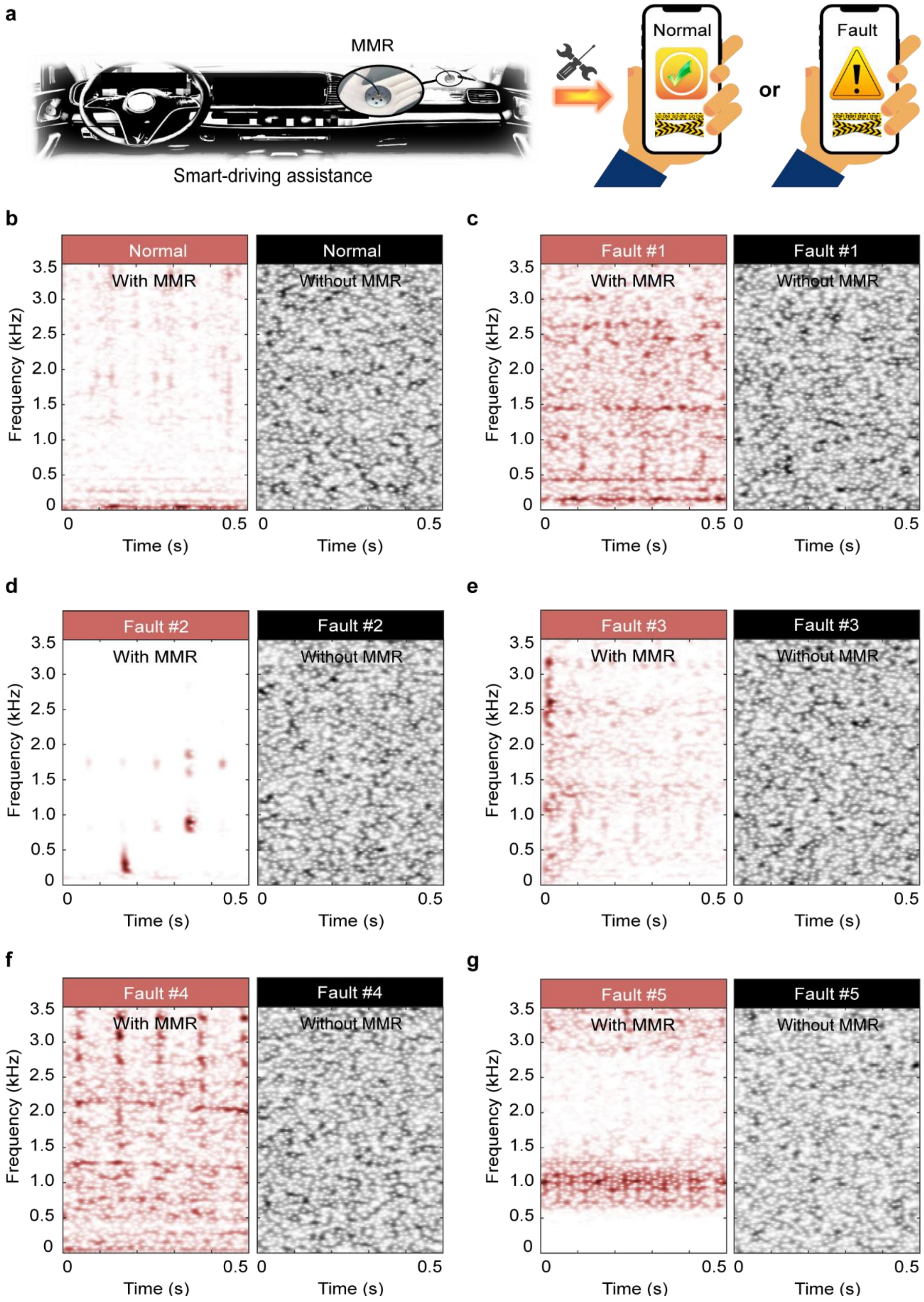

Supplementary Fig. 20 | Details of signal measurements in smart-driving assistance. a, Schematic of smart-driving assistance by equipping MMR into the in-vehicle system. The measured vibration signals from the engine, including one normal signal and five fault signals (Fault #1-#5). The signal measurements corresponding to (**b**) "Normal", (**c**) "Fault #1", (**d**) "Fault #2", (**e**) "Fault #3", (**f**) "Fault #4" and (**g**) "Fault #5" with and without MMR.

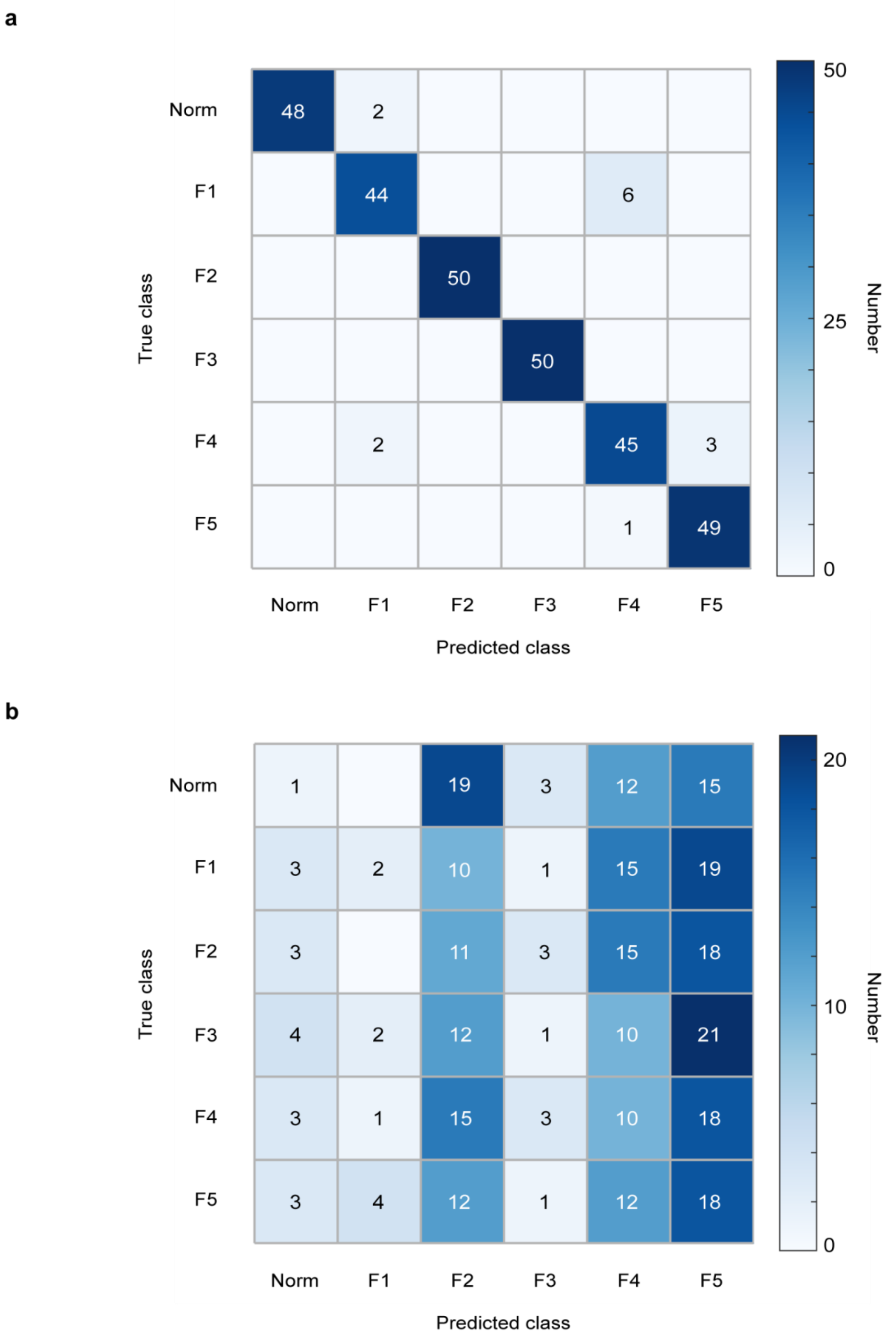

Supplementary Fig. 21 | Recognition comparison with and without MMR in smart-driving assistance. a, The average recognition accuracy with MMR is 95.3%. **b**, The average recognition accuracy without MMR is 14.3%.

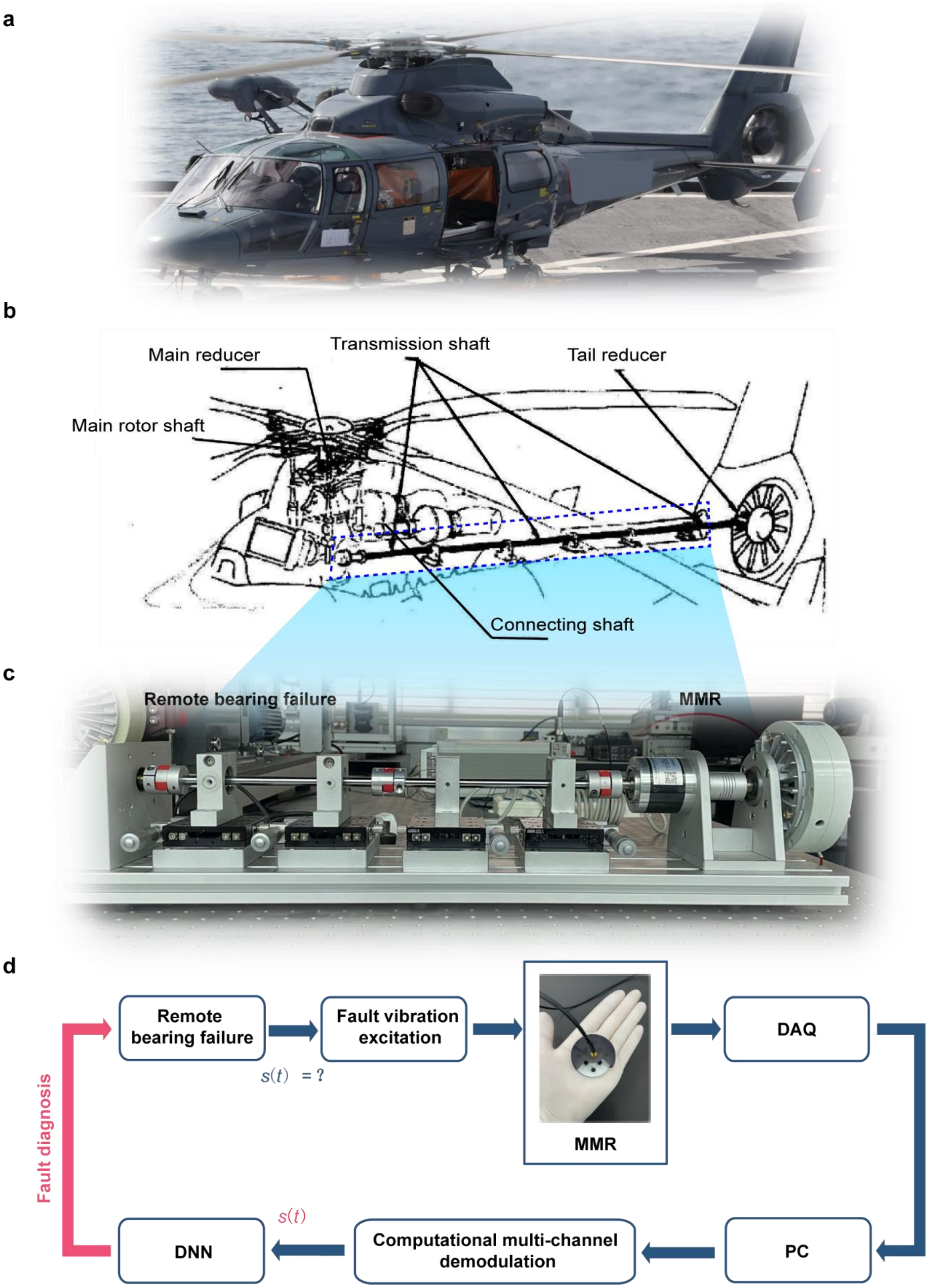

Supplementary Fig. 22 | Demonstration of structural health monitoring. **a**, Helicopter. **b**, Helicopter drive system with slender tail shaft. **c**, Rotor test bench for structural health monitoring of slender tail shaft. **d**, Schematic diagram of the experimental layout.

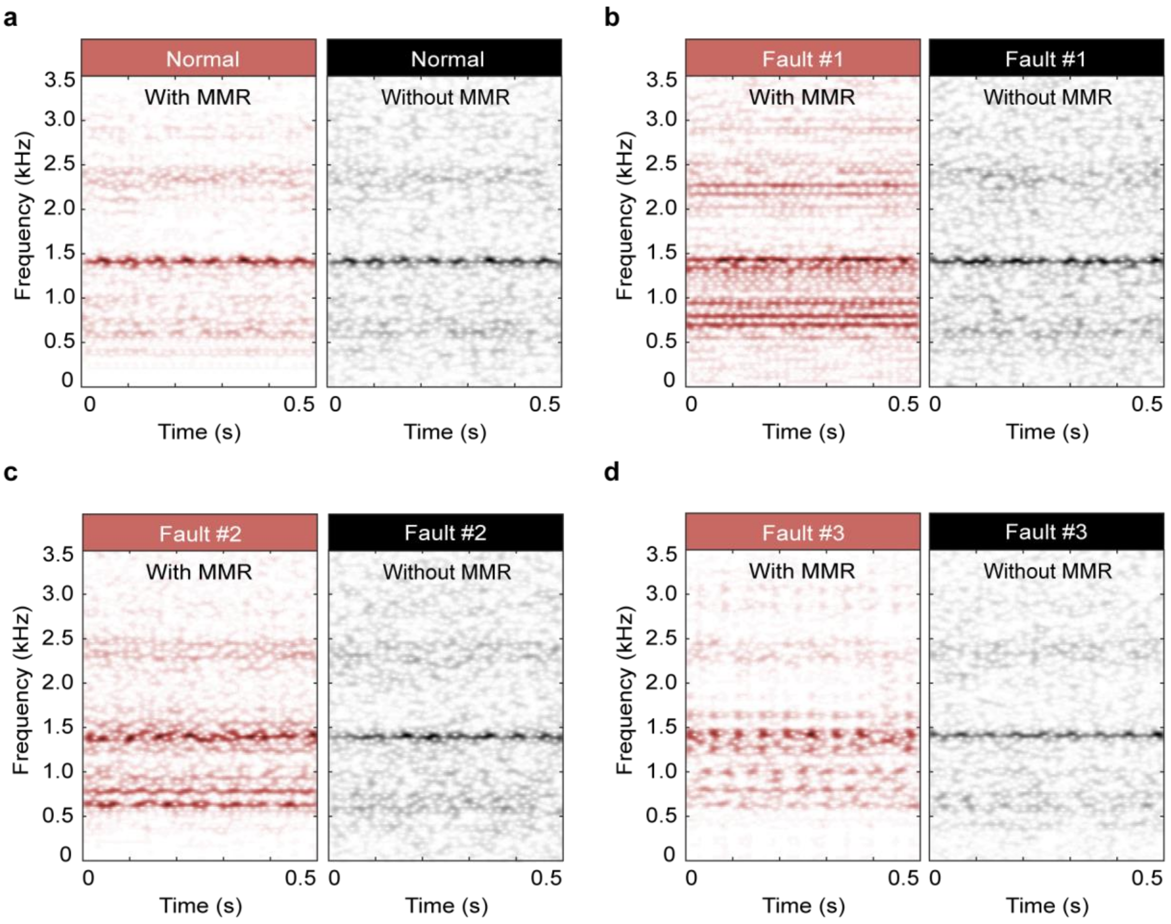

Supplementary Fig. 23 | Details of signal measurements in structural health monitoring. The measured vibration signals from the bear, including one normal signal and three fault signals (Fault #1-#3). The signal measurements corresponding to (**a**) "Normal", (**b**) "Fault #1", (**c**) "Fault #2" and (**d**) "Fault #3" with and without MMR.

a

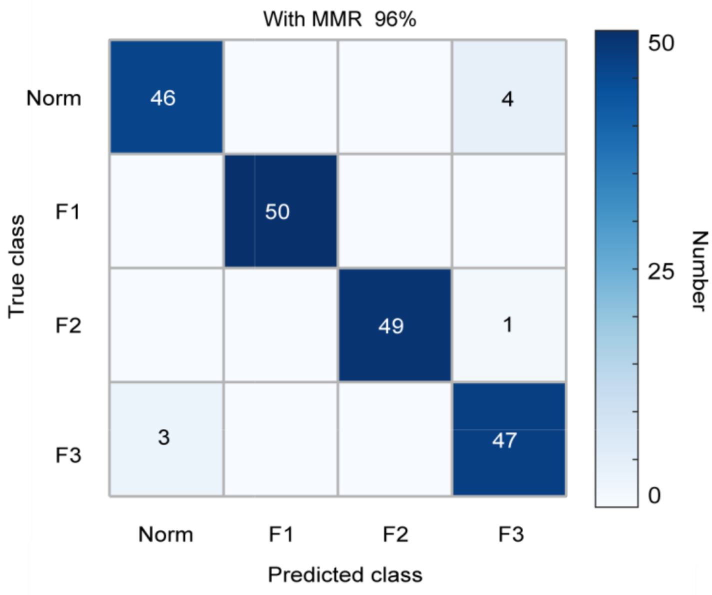

b

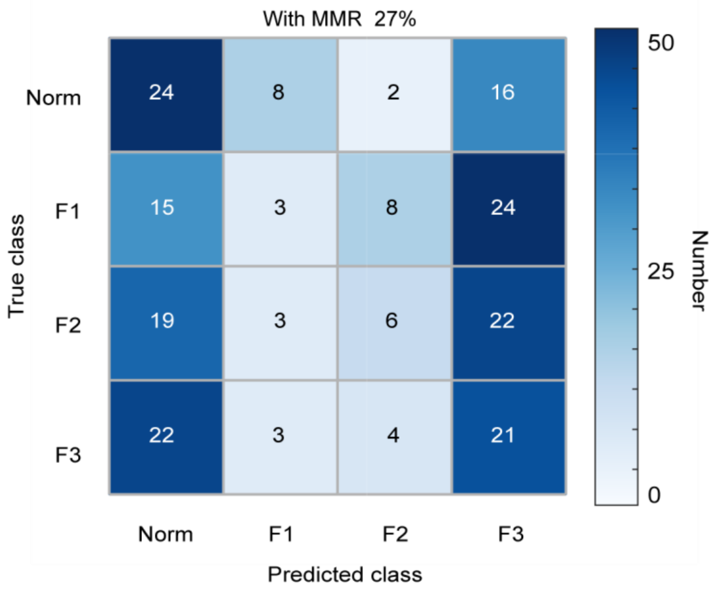

Supplementary Fig. 24 | Recognition comparison with and without MMR in structural health monitoring. a, The average recognition accuracy with MMR is 96%. **b**, The average recognition accuracy without MMR is 27%.

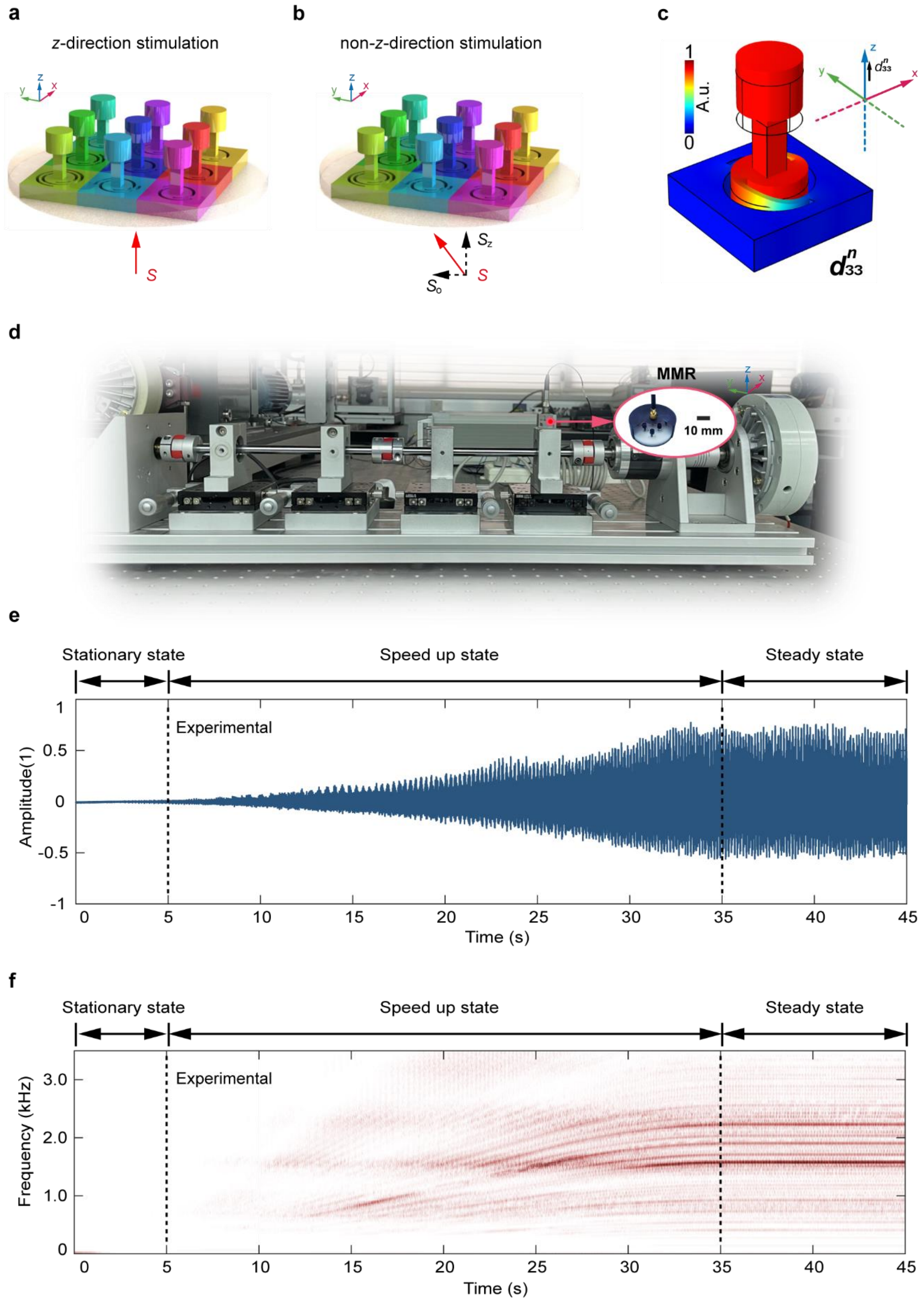

Supplementary Fig. 25 | Running status detection of rotor test bench. a, *z*-direction stimulation. **b**, non-*z*-direction stimulation. **c**, local resonance of unit cell. **d**, rotor test bench. The (**e**) time-domain and (**f**) time-frequency presentation of measured signals during the start-up process of the test bench.

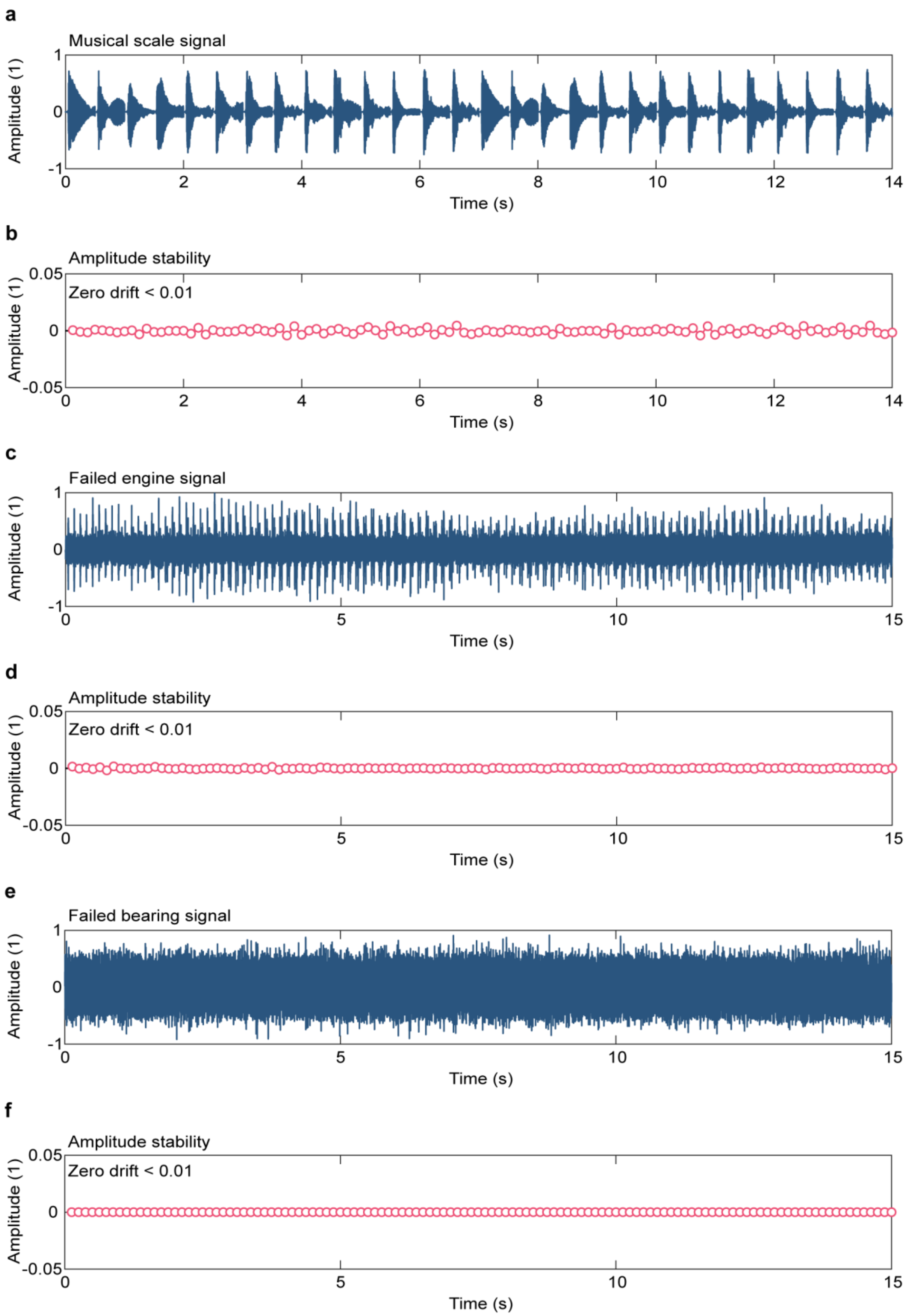

Supplementary Fig. 26 | Specific data on stability and reliability in different application scenarios. a, Musical scale signal and its (**b**) amplitude stability. **c**, Failed engine signal and its (**d**) amplitude stability. **e**, Failed bearing signal and its (**f**) amplitude stability.

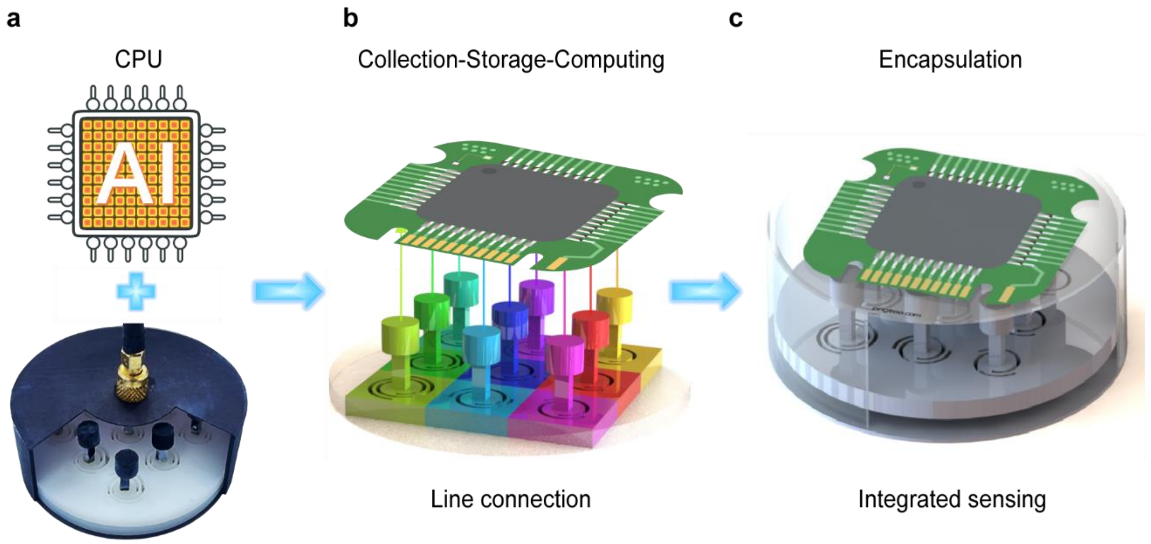

Supplementary Fig. 27 | Integrated sensing of MMR. a, Central processing unit (CPU) and MMR. **b**, CPU integrating data collection, storage and calculation functions. **c**, Schematic diagram of integrated sensing with MMR.

Supplemental Tables

Supplementary Table 1. Structural parameters of the designed unit cells in MMR.

Unit #n	#1	#2	#3	#4	#5	#6	#7	#8	#9
θ_n (rad)	12.70	12.12	11.70	11.25	10.85	10.63	10.50	10.25	10.00

Supplementary Table 2. Dynamic parameters of the mechanical frequency-division multiplexing system.

Unit #*n*	#1	#2	#3	#4	#5	#6	#7	#8	#9
k_n (N/m)	4.54e3	1.12e4	2.22e4	3.71e4	5.66e4	8.05e4	1.08e5	1.47e5	1.98e5
m (kg)	6.0e-4	6.0e-4	6.0e-4	6.0e-4	6.0e-4	6.0e-4	6.0e-4	6.0e-4	6.0e-4
c_n	0.01	0.01	0.01	0.01	0.01	0.02	0.025	0.03	0.04

Supplementary Table 3. Comparisons of bandwidth between the resonant sensors and our work

Methods	Ref.[9]	Ref.[10-11]	Ref.[12]	Ref.[13]	Ref.[14-15]	Ref.[16]	Ref.[17]	Ref.[18]	**Our work**
Band-width	3.5 Hz	5 Hz	100 Hz	320 Hz	500 Hz	1 kHz	1.2 kHz	1.5 kHz	**12 kHz**

Supplementary Table 4. Comparisons between the bioinspired sensors and our work

	Bio-inspired	Function	Sensitivity	Bandwidth
Ref.[19]	Human cochlea-inspired	Acoustic sensing	Piezoelectric coefficient of 46 pC N^{-1}	1 kHz
Ref.[20]	Spider crack-Inspired	Vibration sensing	100-fold improvement	1 kHz
Ref.[21]	Human skin-inspired	Tactile sensing	Detection limit of 0.1 kPa	1 kHz
Ref.[22]	Chameleon skin-inspired	Vibration sensing	610 mW output power under 0.245% strain	5 Hz
Ref.[23]	Scorpion crack-Inspired	Strain sensing	Gauge factor of 1344.1 at 200% strain	No consideration
Ref.[24]	Human skin-inspired	Strain sensing	24-fold improvement	No consideration
Ref.[25]	Mammalian whisker-inspired	Pressure sensing	8% Pa^{-1}	No consideration
Ref.[26]	Mimosa-Inspired	Pressure sensing	50.17 kPa^{-1}	No consideration
Ref.[27]	Human skin-inspired	Pressure sensing	8.5 kPa^{-1}	No consideration
Ref.[28]	Fish scale-Inspired	Pressure sensing	70.86% kPa^{-1}	No consideration
**Our work**	**Rat vibrissae-inspired**	**Micro-motion Sensing**	**Two orders of magnitude improvement (24930 pC N^{-1})**	**12 kHz**

Supplementary Table 5. Frequency parameters of signal components in spatio-temporal sensing.

Time (s)	0.1	0.2	0.3	0.4	0.5	0.6	0.7	0.8	0.9
“S”(Hz)	900	650	400	1200	1500	1800	2900	2500	2100
“J” (Hz)	400	650	900	650	1500	2500	2100	0	0
“T”(Hz)	400	650	900	650	1500	2500	0	0	0
“U”(Hz)	400	1200	2100	2500	2900	1800	900	0	0

Supplementary References

1 Liu, Z. Locally Resonant Sonic Materials. *Science* **289**, 1734-1736, doi:10.1126/science.289.5485.1734 (2000).

2 Bonacchini, G. E. & Omenetto, F. G. Reconfigurable microwave metadevices based on organic electrochemical transistors. *Nature Electronics* **4**, 424-428, doi:10.1038/s41928-021-00590-0 (2021).

3 Graff, K. F. Wave motion in elastic solids. *Dover, New York* (1991).

4 P. M. Morse, H. F. Methods of Theoretical Physics. *McGraw-Hill, New York* (1953).

5 Qiu, C. *et al.* Transparent ferroelectric crystals with ultrahigh piezoelectricity. *Nature* **577**, 350-354, doi:10.1038/s41586-019-1891-y (2020).

6 Eringen, A. C. Microcontinuum field theories: I. Foundations and solids. *Springer* (1999).

7 Cui, H. *et al.* Three-dimensional printing of piezoelectric materials with designed anisotropy and directional response. *Nat Mater* **18**, 234-241, doi:10.1038/s41563-018-0268-1 (2019).

8 Cui, H. *et al.* Design and printing of proprioceptive three-dimensional architected robotic metamaterials. *Science* **376**, 1287-1293, doi:10.1126/science.abn0090 (2022).

9 Pandit, M. *et al.* Closed-Loop Characterization of Noise and Stability in a Mode-Localized Resonant MEMS Sensor. *IEEE Trans Ultrason Ferroelectr Freq Control* **66**, 170-180, doi:10.1109/TUFFC.2018.2878241 (2019).

10 Pandit, M. *et al.* An Ultra-High Resolution Resonant Mems Accelerometer. *Proc Ieee Micr Elect*, 664-667, doi:10.1109/MEMSYS.2019.8870734 (2019).

11 Sobreviela-Falces, G. *et al.* A Mems Vibrating Beam Accelerometer for High Resolution Seismometry and Gravimetry. *2021 34th Ieee International Conference on Micro Electro Mechanical Systems*, 196-199, doi:10.1109/Mems51782.2021.9375431 (2021).

12 Xudong, Z., Thiruvenkatanathan, P. & Seshia, A. A. A Seismic-Grade Resonant MEMS Accelerometer. *Journal of Microelectromechanical Systems* **23**, 768-770, doi:10.1109/jmems.2014.2319196 (2014).

13 Pandit, M., Zhao, C., Sobreviela, G., Zou, X. & Seshia, A. A High Resolution Differential Mode-Localized MEMS Accelerometer. *Journal of Microelectromechanical Systems* **28**, 782-789, doi:10.1109/jmems.2019.2926651 (2019).

14 Shin, D. D. *et al.* Environmentally Robust Differential Resonant Accelerometer in a Wafer-Scale Encapsulation Process. *30th Ieee International Conference on Micro Electro Mechanical Systems*, 17-20, doi:10.1109/MEMSYS.2017.7863328 (2017).

15 Yin, Y., Fang, Z., Liu, Y. & Han, F. Temperature-Insensitive Structure Design of Micromachined Resonant Accelerometers. *Sensors (Basel)* **19**, doi:10.3390/s19071544 (2019).

16 Miani, T. *et al.* Nanoresonator-based accelerometer with large bandwidth and improved bias stability. *Int Symp Inert Senso*, doi:10.1109/Inertial53425.2022.9787526 (2022).

17 Xu, L., Wang, S., Jiang, Z. & Wei, X. Programmable synchronization enhanced MEMS resonant accelerometer. *Microsyst Nanoeng* **6**, 63, doi:10.1038/s41378-020-0170-2 (2020).

18 Miani, T. *et al.* Resonant Accelerometers Based on Nanomechanical Piezoresistive Transduction. *2021 34th Ieee International Conference on Micro Electro Mechanical Systems*, 192-195, doi:10.1109/Mems51782.2021.9375287 (2021).

19 Yan, W. *et al.* Single fibre enables acoustic fabrics via nanometre-scale vibrations. *Nature* **603**, 616-623, doi:10.1038/s41586-022-04476-9 (2022).
20 Fratzl, P. & Barth, F. G. Biomaterial systems for mechanosensing and actuation. *Nature* **462**, 442-448, doi:10.1038/nature08603 (2009).
21 Chun, S. *et al.* An artificial neural tactile sensing system. *Nature Electronics* **4**, 429-438, doi:10.1038/s41928-021-00585-x (2021).
22 Chen, X. *et al.* Bio-inspired flexible vibration visualization sensor based on piezo-electrochromic effect. *Journal of Materiomics* **6**, 643-650, doi:10.1016/j.jmat.2020.06.002 (2020).
23 Sun, H. *et al.* A Highly Sensitive and Stretchable Yarn Strain Sensor for Human Motion Tracking Utilizing a Wrinkle-Assisted Crack Structure. *ACS Appl Mater Interfaces* **11**, 36052-36062, doi:10.1021/acsami.9b09229 (2019).
24 Jiang, Y. *et al.* Auxetic Mechanical Metamaterials to Enhance Sensitivity of Stretchable Strain Sensors. *Adv Mater* **30**, e1706589, doi:10.1002/adma.201706589 (2018).
25 Takei, K. *et al.* Highly sensitive electronic whiskers based on patterned carbon nanotube and silver nanoparticle composite films. *Proc Natl Acad Sci U S A* **111**, 1703-1707, doi:10.1073/pnas.1317920111 (2014).
26 Su, B., Gong, S., Ma, Z., Yap, L. W. & Cheng, W. Mimosa-inspired design of a flexible pressure sensor with touch sensitivity. *Small* **11**, 1886-1891, doi:10.1002/smll.201403036 (2015).
27 Tang, H., Nie, P., Wang, R. & Sun, J. Piezoresistive electronic skin based on diverse bionic microstructure. *Sensors and Actuators A: Physical* **318**, doi:10.1016/j.sna.2020.112532 (2021).
28 Wang, J. *et al.* Bionic Fish-Scale Surface Structures Fabricated via Air/Water Interface for Flexible and Ultrasensitive Pressure Sensors. *ACS Appl Mater Interfaces* **10**, 30689-30697, doi:10.1021/acsami.8b08933 (2018).
29 Bao, P., She, L., McGill, M. & Tsao, D. Y. A map of object space in primate inferotemporal cortex. *Nature* **583**, 103-108, doi:10.1038/s41586-020-2350-5 (2020).
30 Jung, Y. H. *et al.* A wireless haptic interface for programmable patterns of touch across large areas of the skin. *Nature Electronics* **5**, 374-385, doi:10.1038/s41928-022-00765-3 (2022).
31 Massari, L. *et al.* Functional mimicry of Ruffini receptors with fibre Bragg gratings and deep neural networks enables a bio-inspired large-area tactile-sensitive skin. *Nature Machine Intelligence* **4**, 425-435, doi:10.1038/s42256-022-00487-3 (2022).
32 Shao, Y., Hayward, V. & Visell, Y. Spatial patterns of cutaneous vibration during whole-hand haptic interactions. *Proc Natl Acad Sci U S A* **113**, 4188-4193, doi:10.1073/ pnas.1520866113 (2016).
33 Contreras-Castillo, J., Zeadally, S. & Guerrero-Ibanez, J. A. Internet of Vehicles: Architecture, Protocols, and Security. *IEEE Internet of Things Journal* **5**, 3701-3709, doi:10.1109/jiot.2017.2690902 (2018).
34 Eiben, A. E. & Smith, J. From evolutionary computation to the evolution of things. *Nature* **521**, 476-482, doi:10.1038/nature14544 (2015).
