# Supplementary material for: Highly sensitive and broadband meta-mechanoreceptor via mechanical frequency-division multiplexing
Source: Nat Commun. 2023 Sep 7;14:5482. doi: 10.1038/s41467-023-41222-9 (PMC10482866; doi:10.1038/s41467-023-41222-9)
Supplement: Supplementary file 3 — Description of Additional Supplementary Files [file 41467_2023_41222_MOESM3_ESM.pdf]

Description of Additional Supplementary Files:

Supplementary Video 1: Vibrissae-inspired meta-mechanoreceptor

Supplementary Video 2: Mechanical frequency-division multiplexing

Supplementary Video 3: Computational multi-channel demodulation

Supplementary Video 4: Demonstration of spatio-temporal sensing

Supplementary Video 5: Demonstration of remote-vibration monitoring

Supplementary Video 6: Demonstration of smart-driving assistance
